# Optimizing the Cascade Deep Neural Network Parameters Using an Egret Swarm Optimisation Algorithm: An Application to PID Tuning for the AVR with Shallow Controller

**DOI:** 10.3390/biomimetics11080560

**Published:** 2026-08-06

**Authors:** Masoud Elhawat, Hüseyin Altınkaya

**Affiliations:** Department of Electrical and Electronics Engineering, Karabuk University, Karabuk 78050, Türkiye

**Keywords:** Deep Neural Network (DNN), Egret Swarm Optimization Algorithm (ESOA), Automatic Voltage Regulator (AVR), PID controller

## Abstract

Voltage regulation of synchronous generators remains a significant and complex challenge in the field of engineering, particularly under varying load conditions. Although various control strategies have been applied to Automatic Voltage Regulator (AVR) systems for managing the terminal voltage of synchronous generators, Proportional–Integral–Derivative (PID) controllers continue to be one of the most fundamental and widely used approaches due to their simplicity, reliability, and robust structure. The tuning process, which involves determining the optimal values of the three fundamental parameters of a PID controller—namely the coefficients for the proportional, integral, and derivative terms (*K_P_*, *K_I_*_,_ and *K_D_*)—is essential to achieving the desired controller performance. While tuning can be performed through simple trial-and-error methods, such approaches often fail to yield satisfactory results. In this study, the tuning of a PID controller, which provides automatic voltage regulation for 1 kW stand-alone synchronous generator constructed as a real physical experimental setup, was performed using a novel hybrid method named ESOA-CDNN, which combines the Egret Swarm Optimization Algorithm (ESOA) and a Cascade Deep Neural Network (CDNN). The ESOA is utilized to optimize the number of hidden layer neurons, the weights, and the biases of the CDNN. Furthermore, since the PID controller can be readily implemented through a standard Programmable Logic Controller (PLC) within the proposed approach, there is no need for additional hardware in the control system. The PID controller tuning was conducted using four different methods: tuning via PLC, tuning using CDNN, tuning using CDNN optimized with Particle Swarm Optimization (PSO-CDNN), and the proposed ESOA-CDNN approach. Experimental results demonstrate that the PID controller tuned with the ESOA-DNN method significantly outperformed the others in terms of settling time and overshoot reduction. The experimental results under sudden load application (0–500 W, 0–1000 W, and 0–550 VA) and sudden load rejection (500–0 W, 1000–0 W, and 550–0 VA) demonstrate that the PID controller tuned using the ESOA-DNN method significantly outperformed the others in terms of settling time and overshoot reduction.

## 1. Introduction

### 1.1. Basic Information

The quality and reliability of electrical power systems are fundamentally dependent on maintaining the system frequency and voltage within nominal limits. Any deviation from these nominal values may lead to deterioration in power quality or even damage to electrical equipment. While frequency regulation is achieved through Load Frequency Control (LFC), voltage regulation is ensured by means of an Automatic Voltage Regulator (AVR). AVR is a control device responsible for regulating the terminal voltage of synchronous generators and maintaining voltage variations within prescribed limits. In power systems where synchronous generators operate in parallel, AVR not only controls the generator output voltage but also plays a crucial role in ensuring proper reactive power sharing among generators [1,2,3,4,5].

Under all operating conditions—ranging from no-load to full-load—the AVR adjusts the terminal voltage of the synchronous generator (SG) to its nominal value. This regulation is accomplished by controlling the excitation current supplied to the generator’s field winding. The output voltage control of the SG is therefore achieved through the excitation system and is commonly referred to as excitation control. The excitation system supplies direct current to the field winding located on the rotor of the synchronous generator and constitutes a critical component of the AVR system from a control perspective. The AVR operates as a closed-loop control system whose primary objective is to maintain the generator terminal voltage at the desired reference level. A typical AVR system consists of an amplifier, an exciter, a synchronous generator, and a sensor [6,7,8,9].

### 1.2. Literature Review and Research Background

Regulation of the terminal voltage in synchronous generators (SGs) has long been recognized as a complex problem in both academic studies and industrial practice. This challenge mainly stems from the inherent nonlinear characteristics of SGs and their operating environments, along with the presence of uncertain and time-varying parameters that significantly affect system performance. The standard AVR benchmark used in the literature is usually modeled through amplifier, exciter, generator, and sensor transfer functions. Although this transfer-function representation is convenient for controller comparison, the AVR remains a challenging control problem because its response is affected by uncertain gains and time constants, excitation limits, nonlinear field dynamics, sensor noise, disturbance inputs, and implementation constraints. For this reason, the research trend has moved from classical fixed-gain PID tuning toward metaheuristic optimization, fractional-order control, fuzzy and neuro-fuzzy structures, robust control, model predictive control, sliding-mode control, and real-system validation.

Among the various control techniques proposed for AVR systems, the Proportional–Integral–Derivative (PID) controller remains the most widely adopted owing to its simple design structure, ease of implementation, and robustness to variations in system parameters. However, the optimal tuning of the three gain parameters (*K_P_, K_I_, and K_D_*) remains a challenging optimization problem. Classical tuning methods such as trial-and-error, Ziegler–Nichols (ZN), and Cohen–Coon (CC) involve intensive mathematical computations, are valid only at linearized operating points, and generate undesirable overshoots and long-term oscillations. Although conventional methods provide satisfactory voltage regulation under steady-state operating conditions, their performance may deteriorate during rapid load variations and changing operating points due to the nonlinear characteristics of synchronous generators. The fixed controller parameters may not provide optimal performance for different loading conditions, resulting in increased voltage deviations, overshoot, and longer settling times. Furthermore, conventional analog AVR systems have limited flexibility in terms of parameter adjustment, monitoring, and integration with modern digital control platforms.

The modern optimization-based AVR literature is commonly associated with Gaing’s PSO-based PID controller design, which showed that evolutionary search can tune PID gains more effectively than conventional settings for improving rise time, settling time, overshoot, and steady-state response [10]. This work established the dominant research pattern in which AVR controller design is formulated as an optimization problem over controller parameters. Later studies expanded this line through multi-objective and nature-inspired algorithms, including African buffalo optimization, ant lion optimization, evolutionary algorithms, chaotic PSO, equilibrium optimizer, Archimedes optimizer, improved RUN optimizer, coronavirus herd-immunity optimizer, enhanced Aquila optimizer, hybrid PSO-GWO, and V-Tiger PID tuning [11,12,13,14,15,16,17,18,19,20,21,22,23,24,25,26]. These studies generally report better nominal transient response than classical PID designs. However, the large number of optimizer-based papers also reveals a methodological weakness: performance improvements are often demonstrated on the same simplified AVR benchmark, making it difficult to separate the real controller contribution from the influence of the selected objective function, optimizer settings, and comparison baseline.

Comparative and algorithmic studies demonstrate that a controller optimized for minimum overshoot may not simultaneously provide the best settling time, frequency-domain margin, or robustness under parameter perturbations [27,28,29,30]. Thus, the quality of AVR control design depends not only on the optimization algorithm but also on how the engineering problem is encoded.

Controllers such as PIDD2, RPIDD2-FOPI, TI-lambda-DN-D2N2, and variable-order fuzzy fractional PID can reduce overshoot and settling time in simulations by giving the optimizer a larger parameter space [31,32,33,34,35]. On the other hand, every added derivative, filter, fractional order, or cascade loop increases tuning dimensionality and may reduce transparency for plant engineers. The AVR field therefore faces a trade-off between performance maximization and controller parsimony. A controller with a slightly inferior step-response metric may be more attractive if it is easier to tune, less sensitive to measurement noise, and more robust to plant-parameter uncertainty. This issue is especially important because many power plants still favor simple PID or PI-based regulators due to ease of maintenance and operator familiarity.

Implementation-oriented work remains less common than simulation-based optimization, but it is essential for translating AVR designs into practice. In [36], PID tuning is implemented for a PLC-based AVR system using an adaptive artificial bee colony-fuzzy logic algorithm, MATLAB/Simulink, OPC UA communication, KEPServerEX, TIA Portal, SCADA, and experimental load tests on a micro-hydro power plant setup. This contribution is important because it demonstrates real-time control and monitoring conditions that are usually absent from idealized simulations. Practical AVR deployment must consider sampling time, data communication, measurement noise, hardware limitations, actuator saturation, field-current limits, and operator maintainability.

Many studies use identical nominal transfer-function parameters, which are useful for benchmarking but insufficient for demonstrating industrial readiness. A practical AVR controller should be evaluated under reference-voltage changes, load disturbances, parameter perturbations, measurement noise, actuator saturation, and possible time delay. Real-system or hardware-in-the-loop validation remains particularly valuable because it reveals issues that are often absent from MATLAB-only studies, such as sampling jitter, communication latency, finite word length, and sensor scaling. Therefore, experimental and PLC-based works can be considered an important corrective to the simulation-centered literature [17,22,36].

Meta-heuristic algorithms (MHAs) have gained significant popularity over the last two decades. In the AVR literature, a wide range of MHAs have been successfully applied, including cuckoo search algorithm [37], yellow saddle goatfish algorithm [38], chaotic black widow algorithm [39], nonlinear sine cosine algorithm [40], whale optimization algorithm [41], coyote optimization algorithm [42], kidney-inspired algorithm [43], artificial bee colony algorithm [44], particle swarm optimization algorithm [45,46], tree seed algorithm [47], gray wolf optimization algorithm [48], monarch butterfly optimization [49], grasshopper optimization algorithm [50], ant lion optimizer [51], salp swarm algorithm [52].

In summary, AVR research shows a clear progression from optimized PID tuning to more advanced and practically aware control strategies. PID- and PIDD-type controllers remain relevant because they are simple, transparent, and compatible with industrial practice [10,11,12,13,14,18,19,20,21,22,23,25,26]. Fractional-order and cascaded controllers provide additional dynamic-shaping capability and often outperform integer-order PID designs on nominal benchmarks [15,31,32,34,35,40,42,53]. Fuzzy, neuro-fuzzy, and learning-based approaches improve adaptability and nonlinear handling but require careful separation of controller and optimizer effects [54,55,56,57,58,59]. Robust control, MPC, SMC, excitation-limited design, time-delay modeling, and PLC-based validation represent the most practically important direction because they connect simulation performance with power-system constraints [17,22,36].

### 1.3. Research Gap and Motivation

The reviewed literature shows that Automatic Voltage Regulator (AVR) control has been extensively studied through PID-based controllers, metaheuristic optimization algorithms, fractional-order controllers, fuzzy and neuro-fuzzy structures, robust control, model predictive control, and sliding-mode control. Early and recent studies have demonstrated that optimized PID and modified PID controllers can significantly improve transient-response indices such as rise time, settling time, overshoot, and steady-state error [10,11,12,13,14,18,19,20,21,23,25,26]. Similarly, fractional-order and cascaded controller structures have provided additional flexibility for shaping AVR dynamics and improving nominal performance [2,3,4,10,19,20,22,26,31,35,40,42,53,54,55,56,60,61]. However, despite this considerable progress, several research gaps remain unresolved.

A large portion of the existing AVR literature focuses mainly on nominal simulation models. Many studies use the standard linear AVR benchmark consisting of amplifier, exciter, generator, and sensor transfer functions, while practical limitations such as excitation-voltage constraints, actuator saturation, field-current limits, measurement noise, communication delay, and real-time implementation issues are often ignored. Although some recent studies have considered excitation-voltage limits, real AVR models, time-delayed systems, and PLC-based implementation [17,22,36], these works are still relatively limited compared with the large number of simulation-oriented optimization studies. Therefore, there is a clear need for AVR controller designs that are evaluated not only under ideal reference-tracking conditions but also under realistic operating constraints and uncertainty scenarios.

The literature is dominated by metaheuristic optimization algorithms, but the performance superiority of many proposed methods is not always demonstrated under a common and fair benchmarking framework. Numerous algorithms, including PSO, ant lion optimizer, equilibrium optimizer, Archimedes optimizer, RUN optimizer, coronavirus herd-immunity optimizer, enhanced whale optimization, Aquila optimizer, marine predator algorithm, Harris hawks optimization, and hybrid PSO-GWO, have been applied to AVR controller tuning [10,12,19,20,21]. However, the reported improvement may depend strongly on the selected objective function, parameter settings, number of runs, comparison algorithms, and controller structure. As a result, it is often difficult to determine whether the improvement is mainly due to the optimization algorithm, the controller architecture, or the performance index used during tuning.

In summary, existing studies on AVR systems primarily focus on controller design, parameter optimization algorithms, comparative performance evaluation, controller tuning techniques, analysis methods, and performance indices. These studies typically present block diagrams of uncontrolled, conventionally controlled, and metaheuristic-based controlled AVR systems, together with transient response, step response, and robustness characteristics. Furthermore, pole–zero maps, Bode plots, robustness analyses, and frequency-domain evaluations are commonly included. It is observed that most studies employ the same conventional AVR model and transfer function, with identical or closely similar parameter ranges. Consequently, the main objective of these studies has been to demonstrate the superiority of the proposed metaheuristic optimization method over previously reported approaches in terms of controller performance.

### 1.4. Novelty and Contributions

In this article, the PID tuning of the Automatic Voltage Regulator (AVR) of a real physical power plant is investigated using a cascade Deep Neural Network (CDNN). The tuning process is carried out by means of four different approaches: conventional PLC-based tuning, CDNN, PSO-CDNN, and ESOA-CDNN. Among the various artificial intelligence (AI) techniques, artificial neural networks (ANNs) generally exhibit superior performance in tasks such as learning, association, classification, generalization, feature extraction, optimization, and control. Through this technique, networks construct their own experience based on information obtained from examples, enabling them to make similar decisions for similar situations. From a technical perspective, the primary task of an ANN is to determine an output set corresponding to a given input set.

A key property of artificial neural networks is that they are designed to establish the relationship between input and output data without requiring explicit knowledge of the physical behavior of the real system. For this reason, in the present study, the relationship between the synchronous generator (SG) terminal voltage and the AVR set-point signal is modeled using an ANN. In other words, the ANN is employed to map the relationship between the generator voltage signal and the reference voltage value.

Unlike conventional machine learning applications where the primary objective is prediction accuracy, the objective of this study is the optimization of PID controller parameters for an AVR system. Therefore, the performance of the proposed model is assessed based on control-oriented criteria, including overshoot, settling time, and voltage regulation capability, rather than only prediction metrics. In summary, the main contributions of this article are as follows:

- This article proposes a novel approach for Automatic Voltage Regulator (AVR) controller design. In contrast to existing studies, the proposed method employs an experimental approach specifically developed for the controlled system, rather than utilizing the conventional AVR model and its transfer function. Data-driven estimation of PID controller gains was performed for a PLC-based Automatic Voltage Regulator (AVR) test bench.
- To the best of the authors’ knowledge, this is the first study to employ CDNN and ESOA to model an AVR system and to perform PID control design.
- In order to determine the optimal CDNN structure, a comparative analysis among three different methods is presented. For each ANN structure, the mean squared error is evaluated, and the optimal architecture is selected accordingly.
- This study demonstrates that metaheuristic algorithms can be integrated with shallow controllers, such as PLC-based controllers, and successfully applied in industrial environments.
- The proposed methodology provides a flexible framework for the development of advanced control strategies in both academic research and industrial applications, and it can be extended to hybrid and large-scale power plants.

### 1.5. Paper Organization

This article is organized as follows. The experimental setup and terminal voltage control using PLC-PID controller are described in Section 2. In Section 3, the descriptions of the proposed ESOA-CDNN PID controller approach are provided in detail. Experimental results are demonstrated in Section 4. Finally, conclusions are given in Section 5.

## 2. Experimental Setup

The experimental setup is designed to include a 4 kW asynchronous induction motor, a 1 kW brushed synchronous generator (SG), a frequency converter (FC), two DC power supplies with different output voltages (i.e., 85 V and 24 V), an encoder, a PLC, an analog DC converter, six contactors, and three current transformers (CTs).

Synchronous generator is a three phase, four-wired, 1 kW, 380 V, 2.3 A AC, 1500 rpm, brushed generator having an excitation voltage of 72 V DC and excitation current of 2.1 A DC. Induction motor is a three phase, 4 kW, 380 V, 8.2 A, 1500 rpm. Frequency converter (drive) is a 2.2 kW, ACS355 type. PLC is a S7-1200 1215C DC/DC/DC type. The PLC has 14 digital inputs (DI), 10 digital outputs (DO), 2 analog inputs (AI), 2 analog outputs (AO), 2 PROFINET ports and 125 KB memory. AI/AQ module is SM 1234 AI 4 × 13 bit/AQ 2 × 14 bit, 0–10 V DC. Energy meter module is SM1238 type. Phase-neutral voltages (U_L1-N_, U_L2-N_, U_L3-N_), phase-to-phase voltages (U_L1-2_, U_L2-3_, _UL3-1_), phase current and neutral current (I_L1_) of SG, I_L2_, I_L3_, I_N_), frequency, active powers (P_L1_, P_L2_, P_L3_, P_total_), reactive powers (Q_L1_, Q_L2_, Q_L3_, Q_total_) apparent powers (S_L1_, S_L2_, S_L3_, S_total_), power factors (pF1, pF2, pF3, pF_total_) and some other parameters can be measured using the Energy Meter module. DC converter has 0–10 V DC control input, maximum input voltage of 90 V DC; maximum current is 15 A DC. The encoder is a 1024 ppr, 24 V, rotary, incremental type encoder. Power supplies are 240 W, 220 V AC/24 V DC and 800 W, 220 V AC/0–85 V DC. Current transformers are 5/5 A. Contactors are with coil voltage of 24 V and contact current of 10 A.

**Figure 1:**
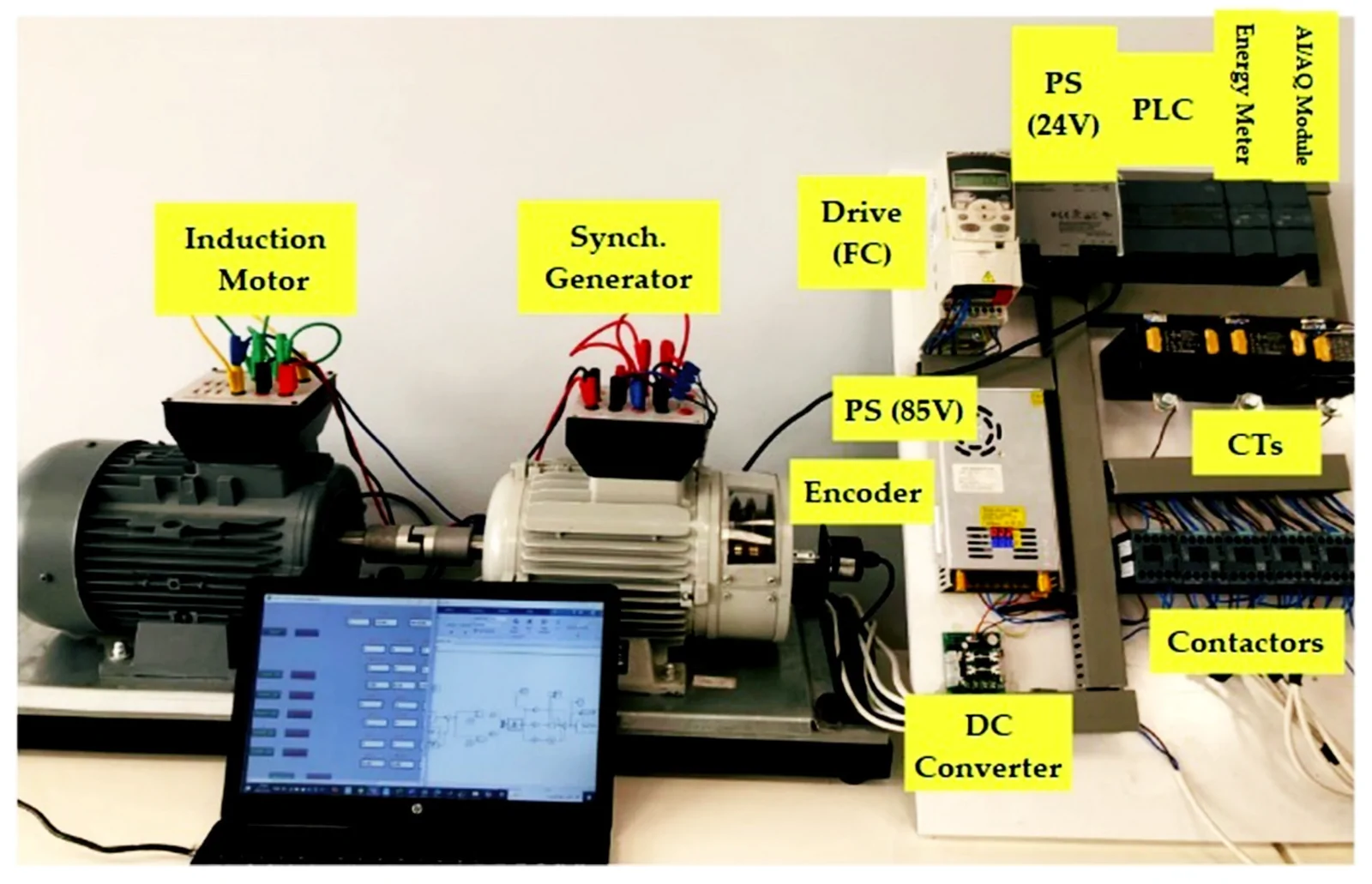
Experimental setup.

The complete layout of the test rig employed in this study is illustrated in Figure 1.

### 2.1. Working Principle of the Experimental Setup

The SG is mechanically driven by an induction motor that is directly coupled to its shaft. The motor is supplied by a frequency converter (FC drive), which adjusts the motor speed and, consequently, controls the output frequency of the SG. The overall operation of the system is controlled by an S7-1215C DC/DC/DC PLC. Thanks to the software developed in the TIA Portal V17 environment and the associated SCADA interface, the system operation can be monitored and controlled in real time.

**Figure 2:**
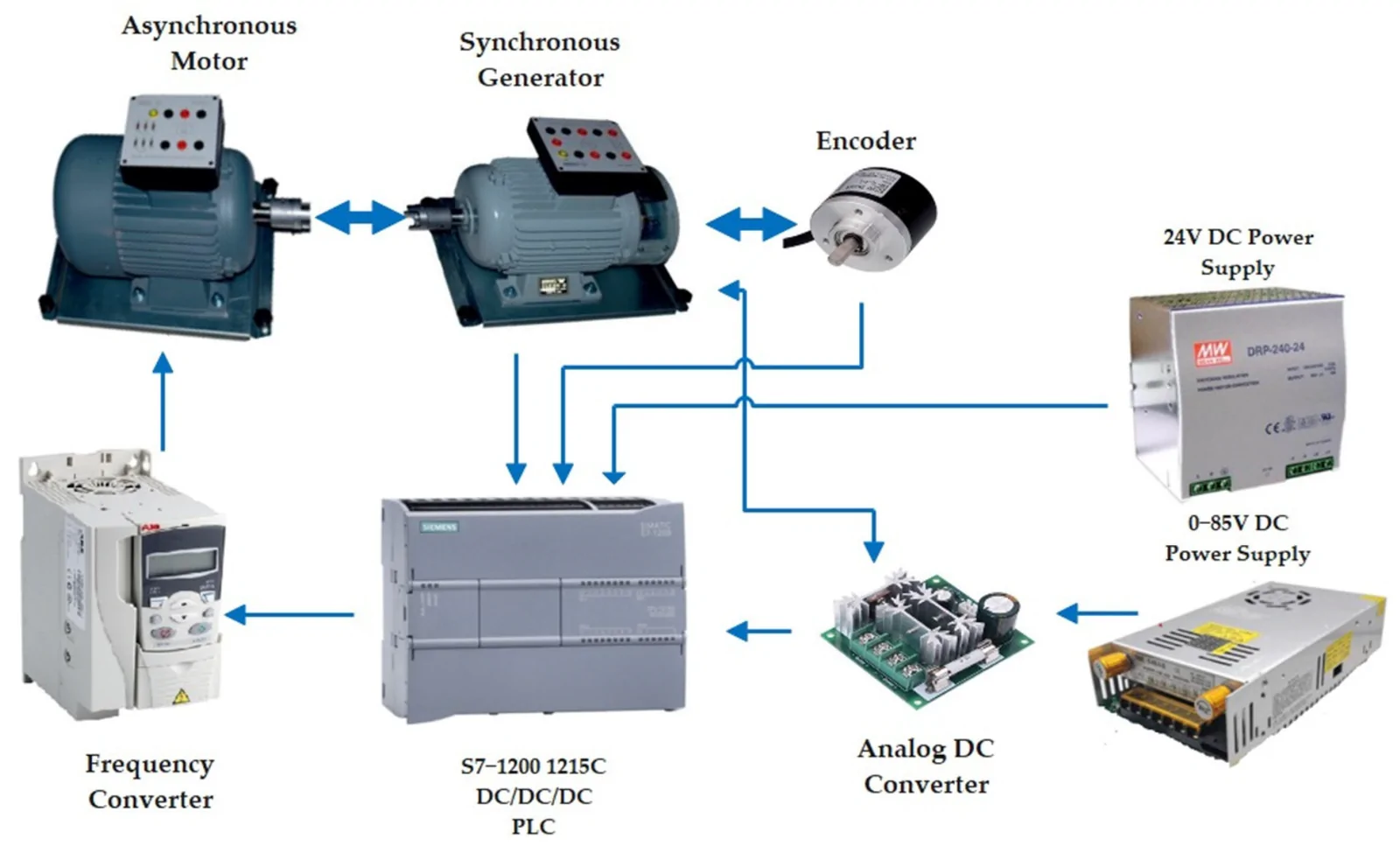
System components.

An analog input/output (AI/AQ) module (SM1234) and an Energy Meter module (SM1238) are integrated into the PLC. The AQ module is responsible for regulating the output frequency and voltage of the synchronous generator by controlling both the drive (frequency converter) and the DC converter, whereas the Energy Meter module continuously measures the electrical parameters, including line-to-line and line-to-neutral voltages, power factor, apparent power, reactive and active power, as well as other related quantities. The PLC and the encoder are supplied from a 24 V DC source, while the rotor windings of the synchronous generator are energized by an independent 0–85 V DC power supply. The current transformers provide an appropriate electrical interface between the synchronous generator and the Energy Meter, and the contactors are used to connect or disconnect different load groups as required. In this configuration, the nominal ratings of the synchronous generator are as follows: 1 kW output power, 380 V AC (220 V line-to-neutral), 50 Hz frequency, 1500 rpm speed, 2.1 A DC excitation current at 72 V DC, and 2.3 A AC line current. A detailed view of all components included in the test setup is presented in Figure 2.

### 2.2. Voltage Control Using the PLC-Based PID Controller

**Figure 3:**
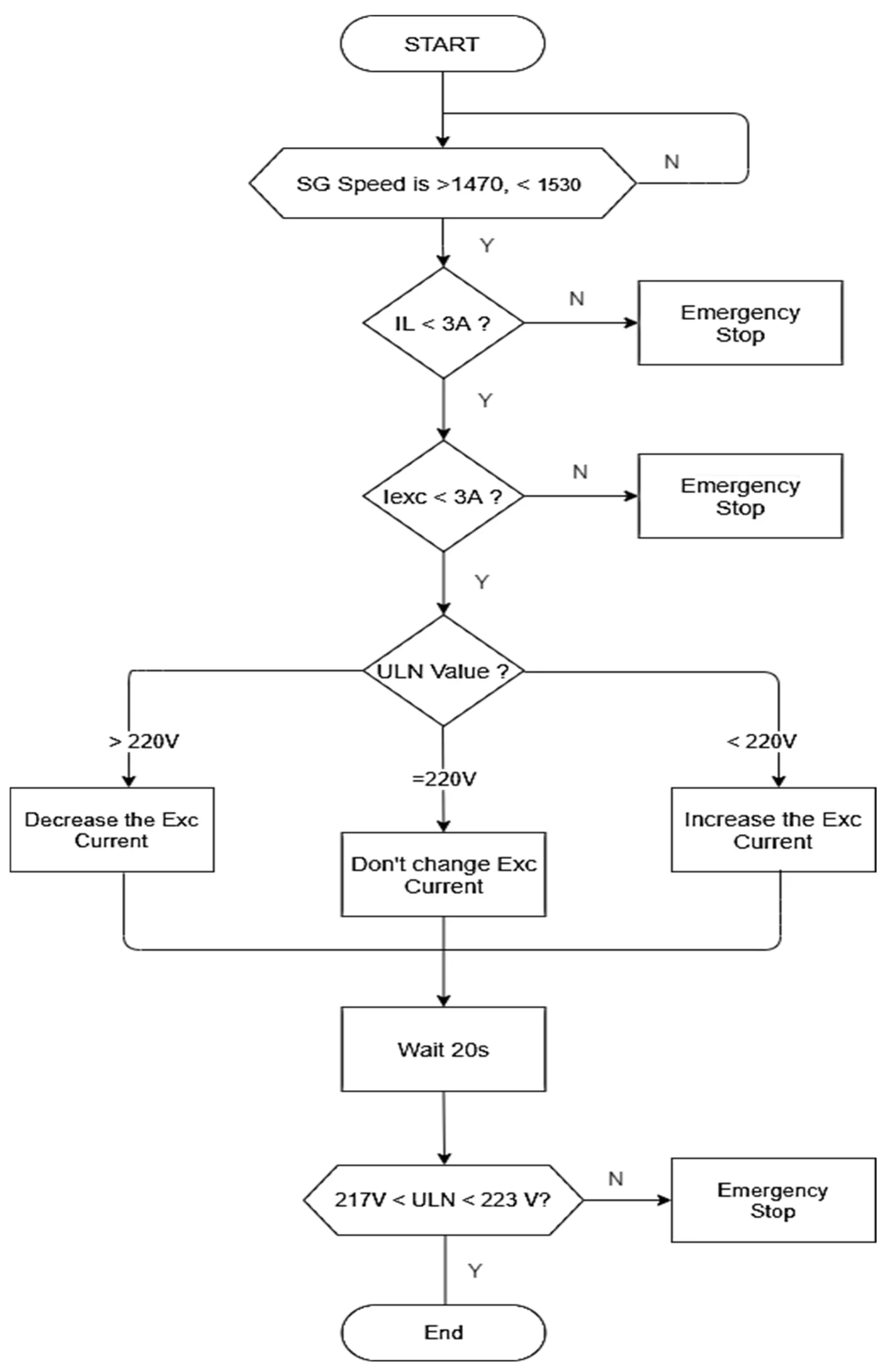
Voltage control flowchart.

The regulation of the synchronous generator (SG) terminal voltage is performed based on the phase-to-neutral voltage measurement ($U_{L2\text{–}N}$) obtained from the Energy Meter. When this measured value deviates from the reference level (220 V), the excitation voltage—and consequently the excitation current is adjusted proportionally. This control action is implemented through a 0–10 V analog signal generated by the PLC analog output and applied to the 0–10 V DC control input of the DC voltage converter. The overall voltage control procedure for the SG is depicted in the flowchart shown in Figure 3.

The regulation of the terminal voltages of synchronous generators (SGs) under varying load conditions is carried out using the PID Compact function. The PID control algorithm operates in accordance with Equation (1).

(1) $$y=K_{P}\left[\left(b.w-x\right)+\frac{1}{T_{I}.s}\left(w-x\right)+\frac{T_{D}.s}{a.T_{D}.s+1}(c.w-x)\right]$$

where y is output value of the PID algorithm, *K_P_* is proportional gain, *s* is Laplace operator, *b* is proportional action weighting, *w* is set point, *x* is process value, *T_I_* is integral action time, *T_D_* is derivative action time, *a* is derivative delay coefficient, *c* is derivative action weighting [62].

**Figure 4:**
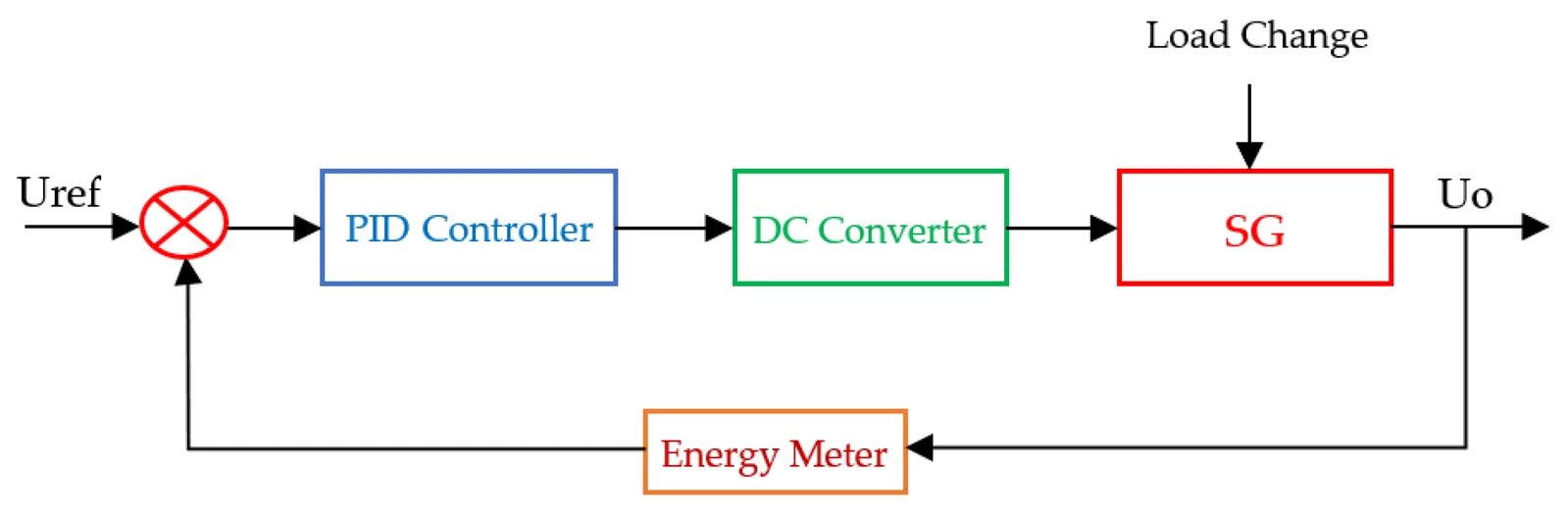
Block diagram of the PID controller used for voltage control via PLC.

**Figure 5:**
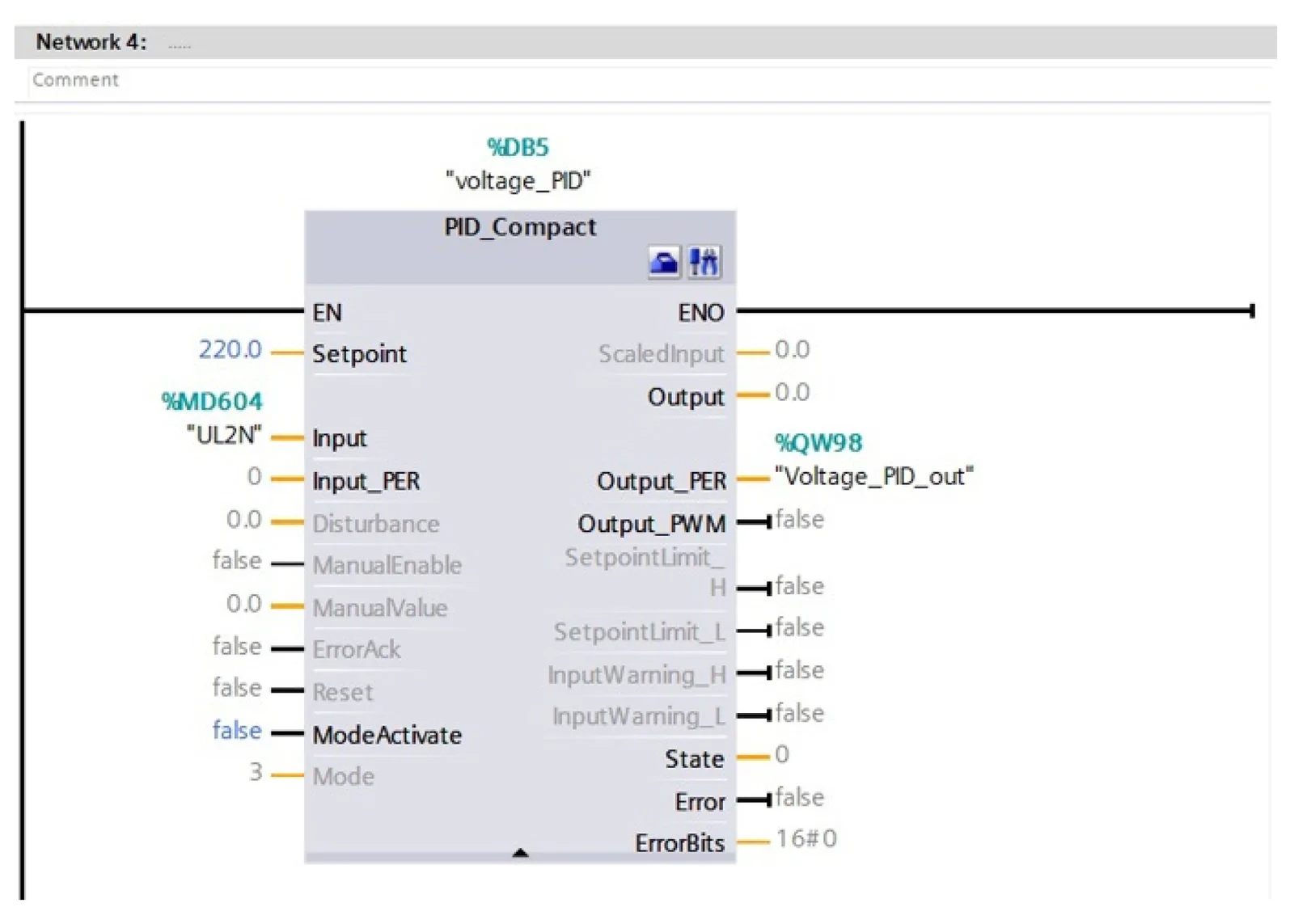
PID compact in TIA portal.

Figure 4 presents the block diagram of the PID-based voltage control implemented via the PLC. The compact PID controller configured for SG within the OB30 Cyclic Interrupt block in the TIA Portal V17 environment is illustrated in Figure 5. PID_Compact acquires the process variable within the control loop and processes it before comparing it with the predefined setpoint. Based on the resulting control error, the controller computes an output value that rapidly converges to the reference value. The output generated by the PID controller is composed of three fundamental components: proportional (P), integral (I), and derivative (D) actions.

**Figure 6:**
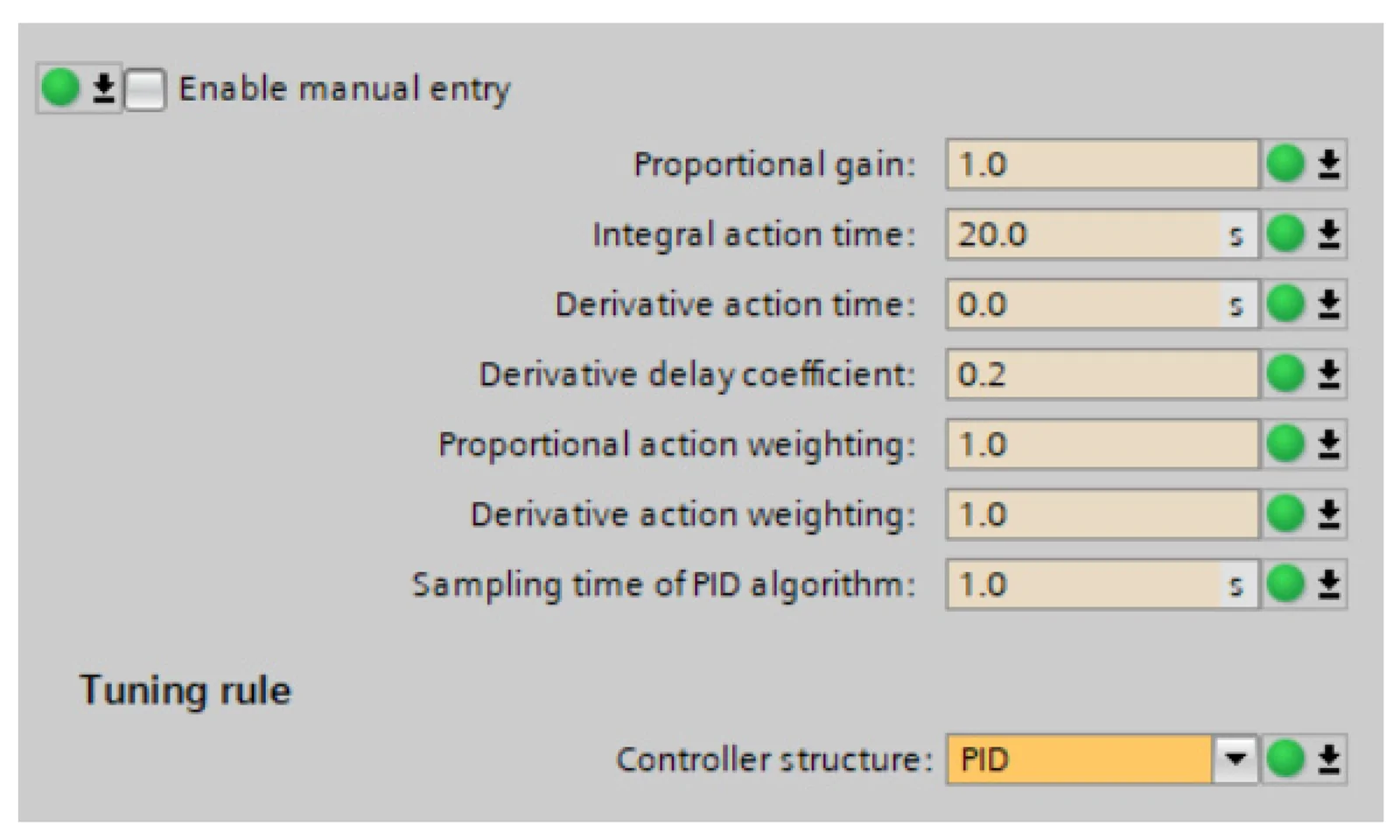
Default (initial) values of PID parameters.

In the configuration of the PID_Compact block, the basic parameters were defined and the controller mode was set to operate in voltage control mode. Within the Input/Output parameters, the signal sources were specified as “Input” and “Output_PER (analog),” since the generator terminal voltage is continuously measured via Energy Meter (SM1238) and analog output of PLC is connected to analog input of DC converter. The voltage setpoint was assigned a value of 220 V. In the PID parameters section, the parameters were left at their default values, as shown in Figure 6. These default values were used as the initial parameter settings.

**Figure 7:**
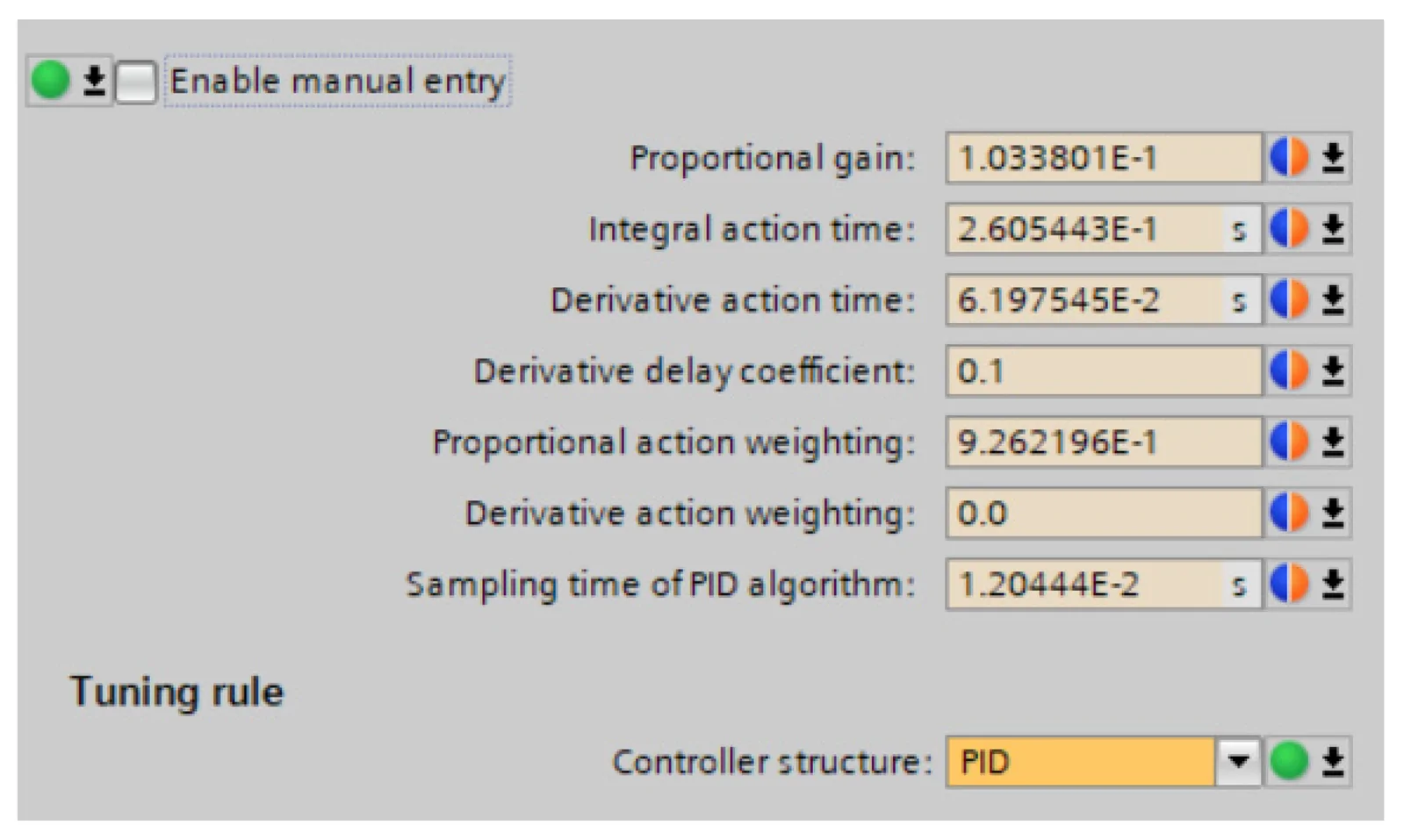
The PID parameter values computed by the PLC.

Once the configuration stage was completed, the real/physical system was commissioned, and the commissioning tool embedded in the PID_Compact block was subsequently utilized to perform the initial auto-tuning procedure followed by fine tuning of the controller parameters. During this procedure, suitable values for $K_{i}$, $K_{p}$, and $K_{d}$ were automatically identified by the optimization algorithm of the controller. Figure 7 illustrates the final tuned parameter values obtained upon completion of both tuning stages.

In summary, the voltage regulation of the synchronous generator (SG) was performed using the S7 1215 PLC-based AVR. Terminal voltage of the SG (UL2-N) was measured by means of the SM1238 Energy Meter module. The measured voltage was compared with the reference voltage of 220 V, and the voltage error was processed by the PLC-based AVR controller. The excitation control signal was generated through the 0–10 V analog output of the PLC (SM1234 AI/AQ module) and applied to the 0–10 V analog control input of the DC voltage clipper circuit (DC–DC converter) connected to the excitation power supply. According to the error signal, the excitation current was increased or decreased proportionally. The analog output signal was linearly scaled to the DC–DC converter input, which regulates the excitation voltage and consequently the excitation current of the field winding. (The DC–DC converter generates an output voltage in the range of 0–90 V, proportional to the 0–10 V DC signal applied to its analog input). The default measurement settings of the SM1238 Energy Meter module and the standard hardware acquisition settings of the SM1234 analog output module were used, and no additional digital filtering or signal smoothing was implemented in the PLC software.

During the experiments, the PLC operated with a minimum cycle time of 1 ms, a maximum cycle time of 8 ms, and a current/last cycle time of 4 ms. The analog output module (SM1234) has a resolution of 14 bits. The sampling time of the PID algorithm was 12 ms. The analog input of the DC–DC converter has a resolution of 13 bits. Experimental parameters and real-time implementation details have been presented in Table 1.

## 3. Proposed ESOA-CDNN Algorithm for PID Controller Tuning

Among the various artificial intelligence (AI) techniques, artificial neural networks (ANNs) generally exhibit superior performance in tasks such as learning, association, classification, generalization, feature extraction, optimization, and control. Through this technique, networks construct their own experience based on information obtained from examples, enabling them to make similar decisions for similar situations. From a technical perspective, the primary task of an ANN is to determine an output set corresponding to a given input set [63,64,65,66].

This section presents a classical Deep Neural Network and ESOA- and PSO-based deep neural networks for tuning PID controller to handle the output voltage fluctuation of SG during loading and unloading. These models aim to find the best hidden layer size and the optimal weights and biases of DNN. The conventional DNN model in developed using MATLAB/NN toolbox; then, the fundamental concepts of ESOA and PSO, as well as the DNN training procedure used to determine the parameter settings, are outlined, followed by the obtained findings.

### 3.1. Deep Neural Network Model

DNN is the foundation of many modern Artificial Intelligence (AI) applications, which consist of multiple hidden layers and neural nodes. A DNN is a distributed processing technique, which allows to save the knowledge learned from of experimental data [67]. While DNN does not require detailed mathematical knowledge being studied, it does need high-quality-trained data to predict the desired output functions. Through the process of training, the algorithm converts the training data to a non-linear mapping between input variables and the output nodes. ANN is typically grouped into feedforward and feedback networks. The former are the most widely used owing to their lower memory requirements practical implementation [68]. Furthermore, they have shown strong performance in modeling non-linear systems, such as a PID tuning. A typical multilayer ANN consists of an input layer, an output layer, and hidden layers.

The neurons in each layer are connected via the weighted neurons and associated bias terms. This distributed processing mechanism can be mathematically expressed as shown in Equation (2):

(2) $$y=\sum_{i=1}^{n}w_{ij}x_{j}+pj$$

where *n* is the number of input signals, xj denotes the input training node, bj is the bias term applied in the hidden and output layers, wij represents the connection weights linking the input, hidden and output layer neurons. To train the feedforward ANN, the backpropagation (BP) algorithm is commonly employed. It is a complex gradient algorithm deployed to enhance ANN performance by adjusting the node weight bias values to minimize the differences between predicted and actual target outputs. During the process, ANN significantly reduces the training error. The mean squared error (MSE) is typically selected as the cost function, as defined in Equation (3):

(3) $$MSE=\frac{1}{n}\sum_{i=1}^{n}\sum_{j=1}^{m}{\left[Y\left(i\right)-T_{j}\left(i\right)\right]}^{2}$$

where m is the number of output signals, *n* is the number of input data, $Yj\;\left(i\right)$ is the actual output, and $Tj\;\left(i\right)$ is the target output. Designing a feedforward DNN typically involves controlling two factors: determining the optimum network topology, mainly the number of hidden layers and the neurons in each layer; and optimizing the initial weights and bias values applied during the training process.

**Figure 8:**
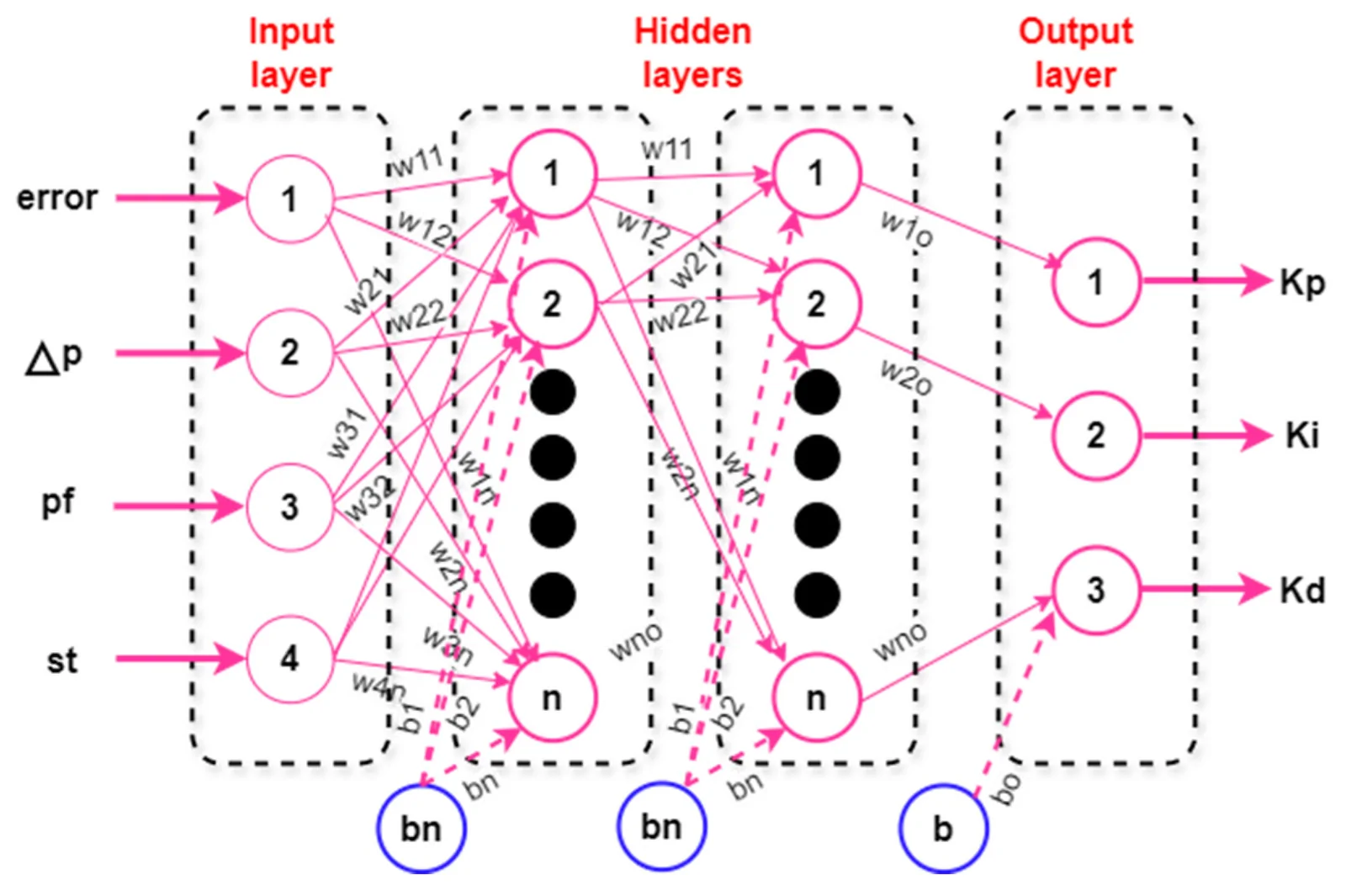
The DNN structure.

For the application of DNN, four parameters which are *e* (error), Δ*P* (change in load), *pf* (power factor of load) and *st* (settling time) were selected as input, while *K_P_, K_I_, and K_D_* were selected as output. The DNN structure is shown in Figure 8.

#### 3.1.1. Hidden Layer Size

Selecting the appropriate hidden layers and neurons is essential for feedforward DNN design, as it directly influences the computational efficiency while maintaining the best regression fit across the ANN node structure [69,70,71,72,73]. If the network contains too many neurons in the hidden layers, the training time significantly extends, and the outputs may suffer from overfitting. Conversely, if the network has too few neurons, it may train quickly, capturing only linear fitting regression, and thus affecting the accuracy. The most common approach to determining the hidden layer is through a trial and error, which is time-consuming and improper for complex models.

#### 3.1.2. Initial Training Weights

As mentioned earlier, the backpropagation (BP) algorithm is employed to train the feedforward ANN model by iteratively exploring the error surface. This process follows the gradient descent method, updating weights incrementally (ΔW), as described in Equation (4).

(4) $$w_{ji}^{I}\left(t\right)=w_{ji}^{I}\left(t-1\right)+{∆w}_{ji}^{I}\left(t\right)$$

The next and previous assumed training weights are represented by $w_{ji}^{I}\left(t\right)$ and $w_{ji}^{I}\left(t-1\right)$, respectively. During each iteration, the BP algorithm operates in two stages: a forward step, which produces updated outputs, and a backward pass, which calculates the MSE and adjusts the weights according to Equations (3) and (4). This process is repeated until obtaining the optimum weight values for training the ANN model. However, several studies have highlighted that this approach may fail to identify the truly optimized training weights, mainly owing to the selected step of updating size of ΔW [74,75,76,77]. If ΔW is too large, the training may then quickly proceed, producing large fluctuations on the error surface, leading to non-converged optimizing outcomes. Conversely, if ΔW is quite small, training becomes slow, and the network may halt before reaching the global minimum error. Thus, determining the initial weights is a key step to ensure the model is accurately trained and produces reliable predictions.

### 3.2. CDNN-PID Controller Tuning

Cascade-forward neural networks resemble conventional feedforward architectures; however, they differ by incorporating direct connections from the input layer as well as from each preceding layer to all subsequent layers. Similar to standard feedforward networks, cascade-forward structures with two or more layers are capable of approximating any finite input–output mapping to an arbitrary degree of accuracy, provided that a sufficient number of hidden neurons is employed [78]. Cascade neural networks are successfully employed in solving various engineering problems [79,80]. Experimental data collected through extensive experiments under varying loadings were recorded using PLC/SCADA software within the TIA portal. The database was then utilized to train and validate the DNN model. The model was developed via Matlab/NN toolbox interface. Four input parameters were chosen for training; error (e), load variation (ΔP), power factor value (pf), and settling time (st). The target outputs were the corresponding optimal PID gains including *K_P_*, *K_I_*, *and K_D_*.

Initially, while the synchronous generator was operating, the proportional, integral, and derivative gains (*K_P_*, *K_I_*, *and K_D_*) were automatically determined by the PLC (Section 2.2, page X, lines X–Y). Using these PLC-generated PID gains, a large number of loading and unloading experiments were conducted under different combinations of resistive and inductive loads. During these experiments, the PLC/SCADA system recorded the error (e), load variation (ΔP), power factor (pf), and settling time (st).

Subsequently, additional datasets were generated by manually assigning various *K_P_*, *K_I_*, *and K_D_* values to the PLC using a trial-and-error procedure. Numerous loading and unloading experiments were then repeated with different PID gain combinations, and the corresponding values of e, ΔP, pf, and st were recorded, thereby increasing the size and diversity of the dataset. Consequently, for both the PLC-generated PID gains and the manually assigned PID gains, the resulting settling time (st) and maximum overshoot (represented by the error (e), obtained under different load combinations, were collected. At this stage, *K_P_*, *K_I_*, *and K_D_* can be regarded as the input variables of the system, whereas st and e represent the corresponding output variables.

The resulting experimental dataset was subsequently used to train and evaluate the CDNN, PSO-CDNN, and ESOA-CDNN models. Since the objective of these models is to determine the PID gains that minimize the settling time and error for a given operating condition, the input–output configuration was reversed. Specifically, settling time (st), error (e), load variation (ΔP), and power factor (pf) were used as the input features, whereas *K_P_*, *K_I_*, and *K_D_* were defined as the output targets. In other words, the CDNN-based models were trained to estimate the optimal PID gains capable of achieving the minimum settling time and error for different loading and unloading conditions.

**Figure 9:**
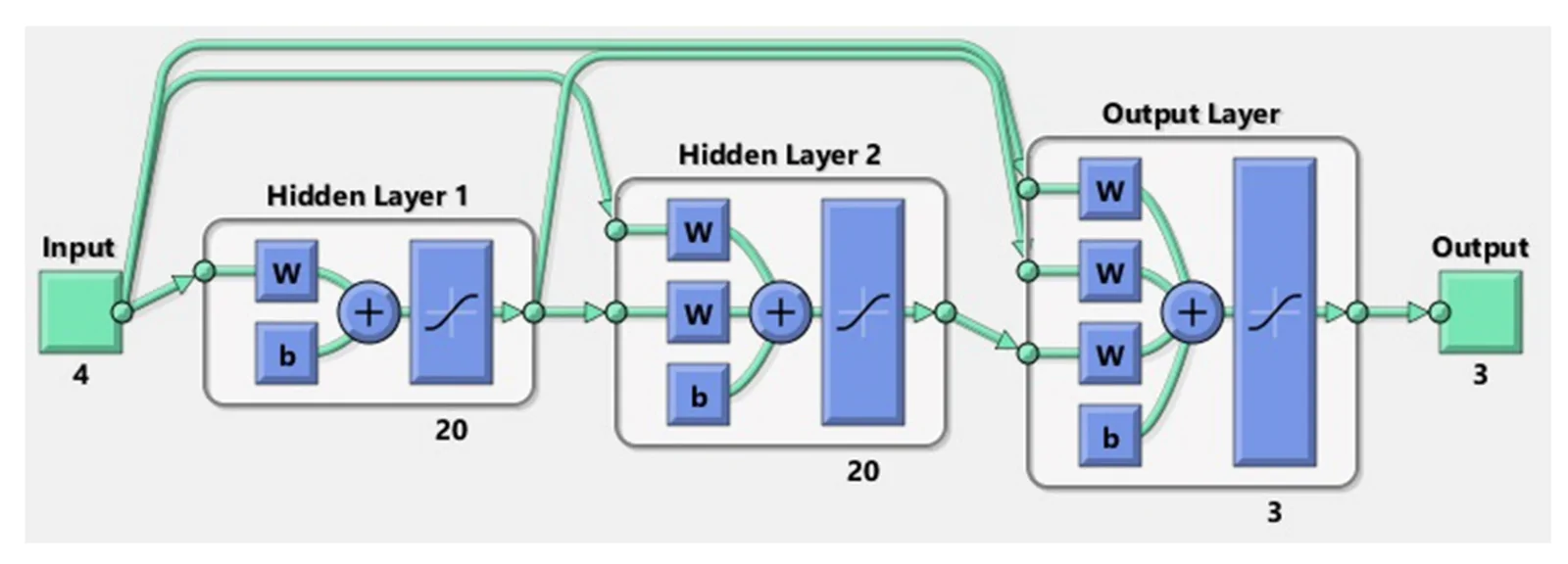
CDNN architecture.

**Figure 10:**
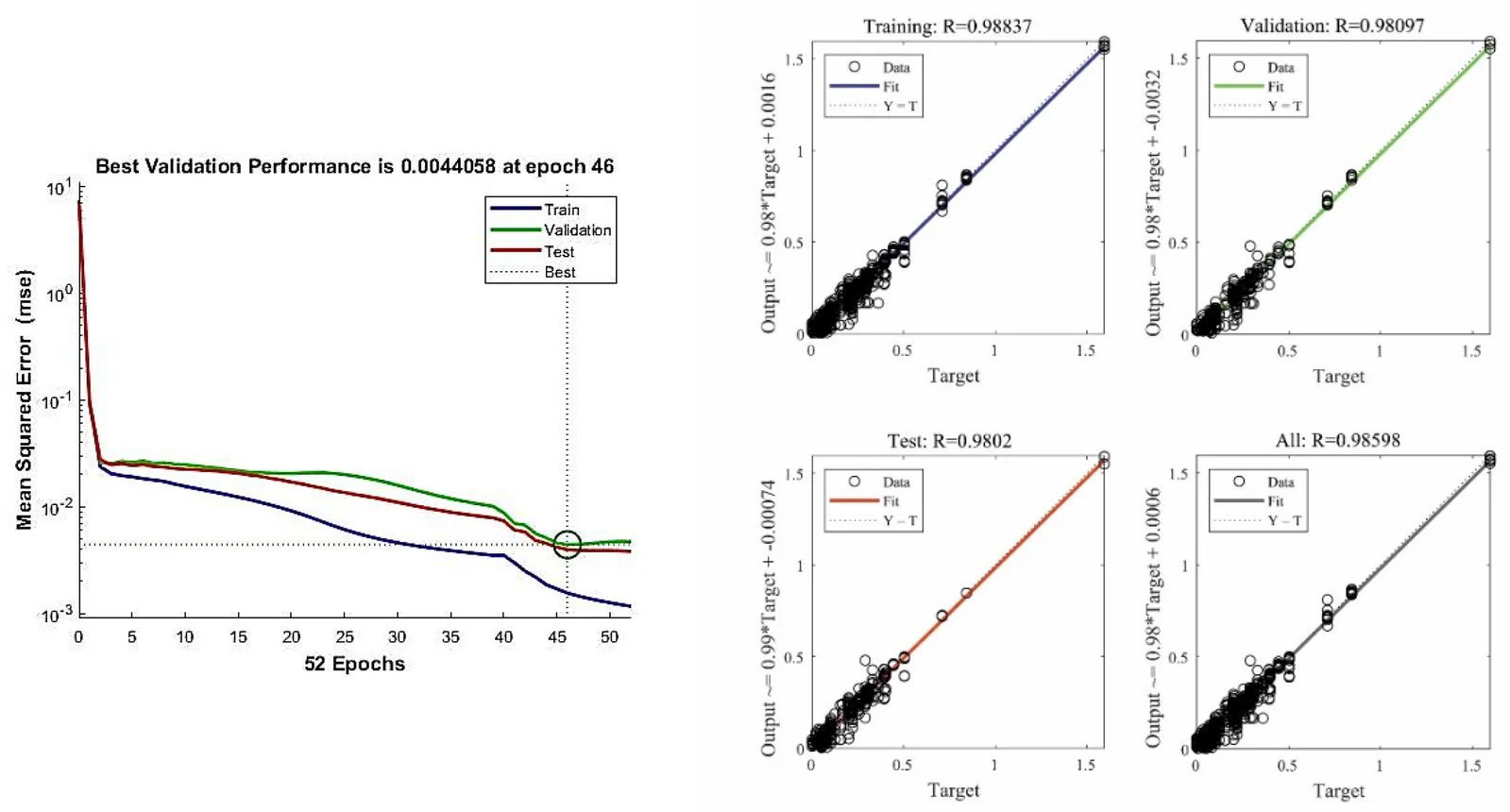
CDNN regression results.

The Cascade-forward architecture with backpropagation learning was employed as transfer function using tansig and the Levenberg–Marquardt as a training model. Among the various CDNNs configurations evaluated, the architecture comprising 20 and 20 neurons in the first and second layer respectively achieved the best findings, as seen in Figure 9. In total, 7 × 768 data samples were utilized to train the network. Figure 10 exhibits the corresponding regression performance findings.

For the CDNN implemented in MATLAB, the following settings were used to ensure reproducibility. The dataset was divided into 70% training, 15% validation, and 15% testing subsets, and normalization was performed using the mapminmax function. The random number generator (RNG) was initialized using rng(42,’twister’). The maximum number of training epochs was set to 250, and early stopping was performed based on the validation performance (min_grad = 1 × 10^−7^, max_fail = 10).

Table 2 summarizes the initial DNN architectures assessed in the current study and their corresponding performance. Each architecture was retrained 35 times to ensure assessment efficiency and minimize the random weight initialization effect on the findings.

### 3.3. Egret Swarm Optimization Algorithm

The Egret Swarm Optimization Algorithm (ESOA), proposed by Zuyan Chen et al. in 2022 [81], is a nature-inspired optimization method derived from the foraging behavior of snowy and great egrets. The algorithm is structured around three fundamental mechanisms: the sit-and-wait strategy, the aggressive strategy, and discriminant conditions. In ESOA, the population is divided into n subgroups, referred to as egret teams, each comprising three individual agents. Within each team, egret A adopts the sit-and-wait approach, whereas egret B and egret C implement aggressive search behaviors through random walk and encirclement mechanisms, respectively.

#### 3.3.1. Sit-And-Wait Strategy

The observation equation of the ith egret *Ai* can be expressed as:

$${\hat{y}}_{i}=E\left(x_{i}\right),$$

where *E* stands for the evaluation function utilized by egret *Ai* to assess potential targets at its current position and ${\;\omega }_{i}$ is the position of group i. During each iteration, the algorithm calculates the predicted prey value of prey ${\hat{y}}_{i}$, updates the parameters of the evaluation function, and determines the corresponding estimation error (ei).

(5) $${\hat{y}}_{i}=\omega_{i}\cdot x_{i}{\hat{y}}_{i}$$

(6) $$ei=\frac{\left|\left|\hat{y}i-\;yi\right|\right|2}{2}$$

$\omega_{i}$ denotes the weight associated with the evaluation function, and the symbol “*” corresponds to elementwise multiplication. The term, ${\hat{g}}_{i}$, represents the practical gradient of $\omega_{i}$, which is computed via the partial derivative of the estimation error ei.

(7) g^i=∂e^i∂ωi=∂|y^i−yi|22∂wi=y^i−yi.xi

(8) $${\hat{g}}_{i}=\frac{{\hat{g}}_{i}}{\hat{\left|g_{i}\right|}}$$

The symbol ${\hat{d}}_{i}$ donates the fight direction of egret of the i-th egret. In the ESOA mechanism, each egrets modifies its movement by considering two different sources of experiential knowledge: the direction correction derived from the best position of egret team ($d_{h,i}$), and the direction correction based on the best position within the entire population ($d_{g,i}$).

(9) $$d_{h,i}=\frac{x_{ibest}-x_{i}}{\left|x_{ibest}-x_{i}\right|}\cdot \frac{f_{ibest}-f_{i}}{\left|x_{ibest}-x_{i}\right|}+d_{ibest}$$

(10) $$d_{g,i}=\frac{x_{gbest}-x_{i}}{\left|x_{gbest}-x_{i}\right|}\cdot \frac{f_{gbest}-f_{i}}{\left|x_{gbest}-x_{i}\right|}+d_{gbest}$$

Here, $d_{ibest}$ represents the flight direction of the highest performing individual within anegret team while $d_{gbest}$ means the flight direction of the best individual across the entire egret population. If a team individual or a population individual finds an improved solution, the corresponding true gradient would be assigned to that individual. $x_{ibest}$ and $x_{gbest}$ are the optimal position values of the egret team and the overall population, respectively, whereas $f_{ibest}$ and $f_{gbest}$ are related to the best fitness values of the team and population. Equations (7)–(10) can be used to produce the integrated gradient $g_{i}$ equation

(11) $$g_{i}=\left(1-r_{h}-r_{g}\right)\cdot {\hat{d}}_{i}+r_{h}\cdot d_{h,i}+r_{g}\cdot d_{g,i}$$

Here, $r_{h}$, $r_{g}$ ∈ $\left[0,\;0.5\right)$.

$\omega_{i}$ is a weight matrix initially generated using a uniform random distribution. During the optimization process,$\;\omega_{i}$ is updated using a random algorithm, as defined by:

(12) $$m_{i}=\beta_{1}\cdot m_{i}+\left(1-\beta_{1}\right)\cdot g_{i}$$

(13) $$v_{i}=\beta_{2}\cdot v_{i}+\left(1-\beta_{2}\right)\cdot {g_{i}}^{2}$$

(14) $$\omega_{i}=\omega_{i}-m_{i}/\sqrt{v_{i}}$$

In the above formula, $\beta_{1}$ is set to 0.9, $\beta_{2}$ is set to 0.99, while the initial values of $m_{i}$ and $v_{i}$ are both assigned as zero.

The updated position of egret A is defined below:

(15) $$x_{a,i}=\;x_{i}\;+\exp\left(\frac{-t}{0.1\cdot t_{max}}\right)\cdot 0.1\cdot hop\cdot g_{i}$$

Here, t denotes the current number, *t_max_* represents the maximum number of iterations, and hop defines the position step size, which is calculated as the difference between the upper and lower bounds of the solution space. Heuristic optimization of controller parameters generally depends on a priori knowledge. For PID controller, the gains kp, ki and kd are initially tuned to reasonable values based on prior experience, with the corresponding feasible ranges used to set the upper and lower limits for the search space.

#### 3.3.2. Aggressive Strategy

Egret B employs a random walk as part of the aggressive strategy. Despite the fact that this approach requires higher energy, it increases the opportunity of locating more rewarding positions.

The position update for egret B is given by the following equation:

(16) $$x_{b,i}=x_{i}+\tan\left(r_{b,i}\right)\cdot \frac{hop}{1-t}$$

where $r_{b,i0}\;$is a random number in $\left(-\frac{\pi }{2},\frac{\pi }{2}\right)$.

Egret C employs the encirclement strategy. Once the prey is identified, the egret initiates chasing it until it is successfully captured.

(17) $$D_{h}=x_{ibest}-x_{i}\;D_{g}=x_{gbest}-x_{i}\;x_{c,i}=\left(1-r_{i}-r_{j}\right)\cdot x_{i}+r_{i}\cdot D_{h}+r_{j}\cdot D_{g}$$

where D_h_ indicates the difference matrix between the current and optimum position of the egret team, while D_g_ refers to the difference matrix between the current and optimal position of egret population. ri and rj are random numbers in [0.5, 0].

#### 3.3.3. Discriminant Condition

After each egret in the group updates its position, the swarm collectively determines the new position of the entire egret population. The resulting matrix is expressed as:

(18) $$x_{s,i}=\left[x_{a,i},x_{b,i},x_{c,i}\right]$$

The egret team compares the updated positions and fitness values of its three egrets with those from the previous iteration. The egret team updated positions and fitness values of the three egrets. If an egret’s new position resulted in improved fitness, it would be adopted; otherwise, a less optimal position has a 30% chance to still be accepted to maintain exploration. To clearly illustrate the procedure clearly, Algorithm 1 details the step-by-step operations of the ESOA. **Algorithm 1:** Egret Swarm Optimization Algorithm**Input:** $x^{0}$**Operation                                                            Cost**/* Strategy Function */1.    **function** SitAndWait(x) 2.        Update the integrated gradient **g** via Equations (4)–(8) 3.          Update the weight of observation method ω by Equations (10)–(12) 4.        Get the expected position $x_{a}$ of EgretA by Equation (9)                                     *T*_1_

5.        Retrieve the Egret A’s fitness $y_{a}$ 6.        **return**
$x_{a}$, $y_{a}$ 7.    **end function** 8.    **function** Aggressive(x) 9.       Get the expected position $x_{b}$ of Egret B by Equation (13) 10.       Get the expected position $x_{c}$ of Egret C by Equation (14) 11.       Retrieve the fitness of Egret B $y_{b}$ and Egret C $y_{c}$
12.       return xb,xc,ya,yc 13.    **end function**/* Discriminant Condition */14.    **while**
*t* < tmax **do**15.         $x_{a}^{t}$, $y_{a}^{t}$ *← SitAndWait* $\left(x^{t}\right)$                                                                                        *T*_2_
16.         
$x_{b}^{t}$, $x_{c}^{t}$, $y_{b}^{t}$, $y_{c}^{t}$ *← Aggressive* $\left(x^{t}\right)$
17.         Get next position x^t+1^ via Equation (15) 18.     **end while**19.     **return**
$x_{best}$, $x_{best}$
/* Operation Ending */**Output:** $x_{best}$, $x_{best}$

### 3.4. ESOA- DNN PID Tuning

**Figure 11:**
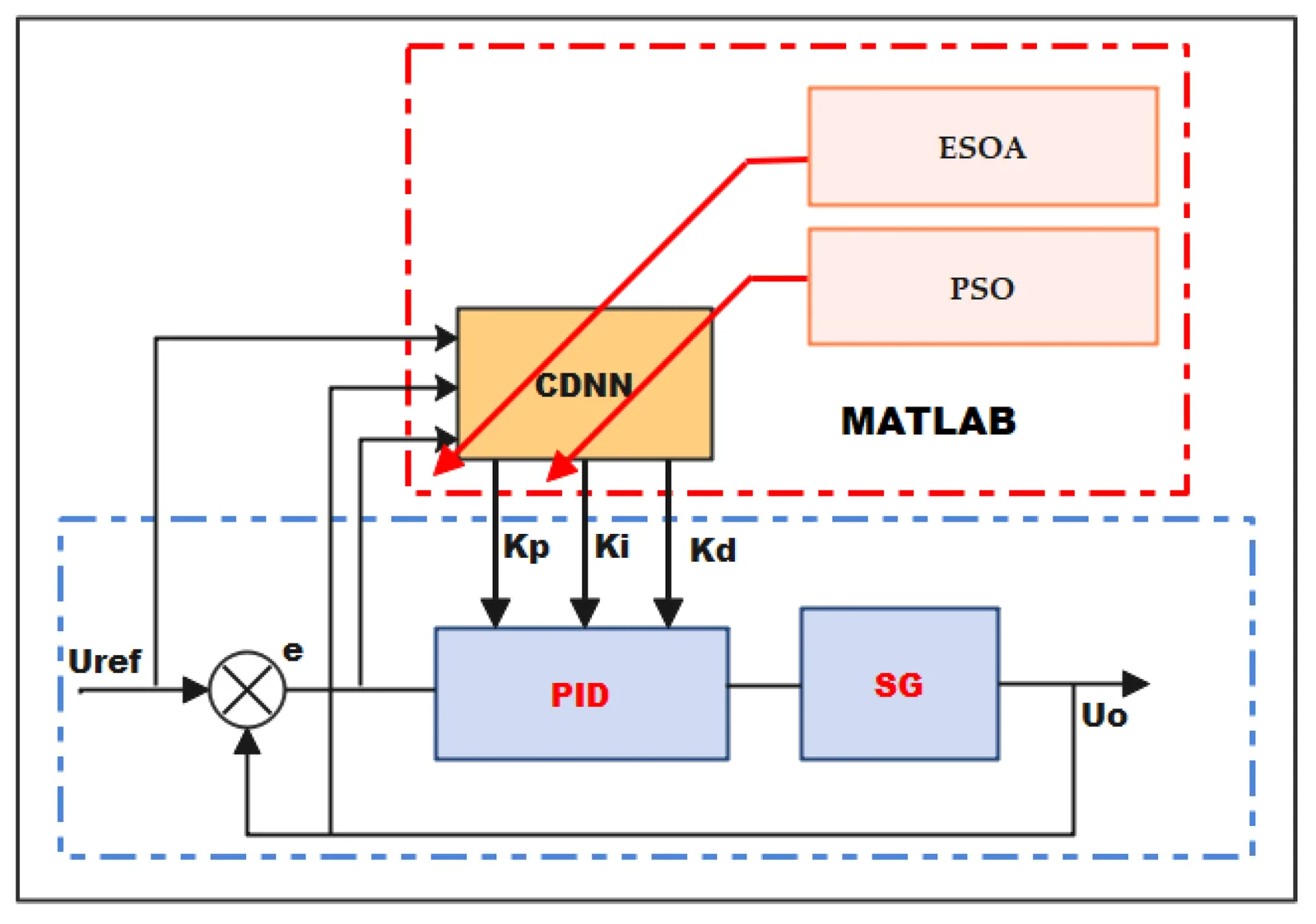
(ESOA, PSO)–CDNN PID tuning.

Using a MATLAB executable code file (.m file), a DNN model relaying on actual database set of a PID tuning is introduced. The input parameters of the DNN approach include error (*e*), load variation (Δ*P*), power factor value (*pf*) and settling time (*st*), while *K_P_*, *K_I_*, *and K_D_* are selected as desired output parameters (targets). The accuracy of *K_P_*, *K_I_*, *and K_D_* using a Cascade Deep Neural Network (CDNN) whose architecture (hidden neurons) and initial weights/biases values are optimized by an evolutionary metaheuristic Egret Swarm Optimization Algorithm (ESOA), then fine-tune the network with Levenberg–Marquardt (LM) for the best accuracy and generalization. MSE is adopted as the object function, and a schematic representation of the training procedure for the ESOA–CDNN algorithm is shown in Figure 11.

#### 3.4.1. ESOA–DNN Algorithm (The Best Architecture of DNN)

In the first stage of this approach, the ESO algorithm is combined with the ANN model to determine the optimal metaheuristic of cascade forward DNN. Cascade forward DNN (CFDNN) was selected because it has direct input connections to deeper layers, often accelerating learning on structured or tabular data and improving approximation quality with similar parameter counts. Thus, a hybrid algorithm is employed to determine the optimal number of neurons in two hidden layers, eliminating the need for the user to preselect the number, which could lead to suboptimal configurations.

In this algorithm, the lower and upper bounds for the numbers of neurons in each hidden layer are set to (4, 0) and (60, 60), respectively. In the present work, a neural network with two hidden layers, four nodes and three output nodes achieved the minimum training error, with the optimized number of neurons in the hidden layers ranging from 6 to 16 neurons. This architecture is employed to identify the optimum initial weights and biases of the DNN model.

#### 3.4.2. ESOA–ANN Algorithm Determining the Initial Weights and Biases of the DNN Model

**Figure 12:**
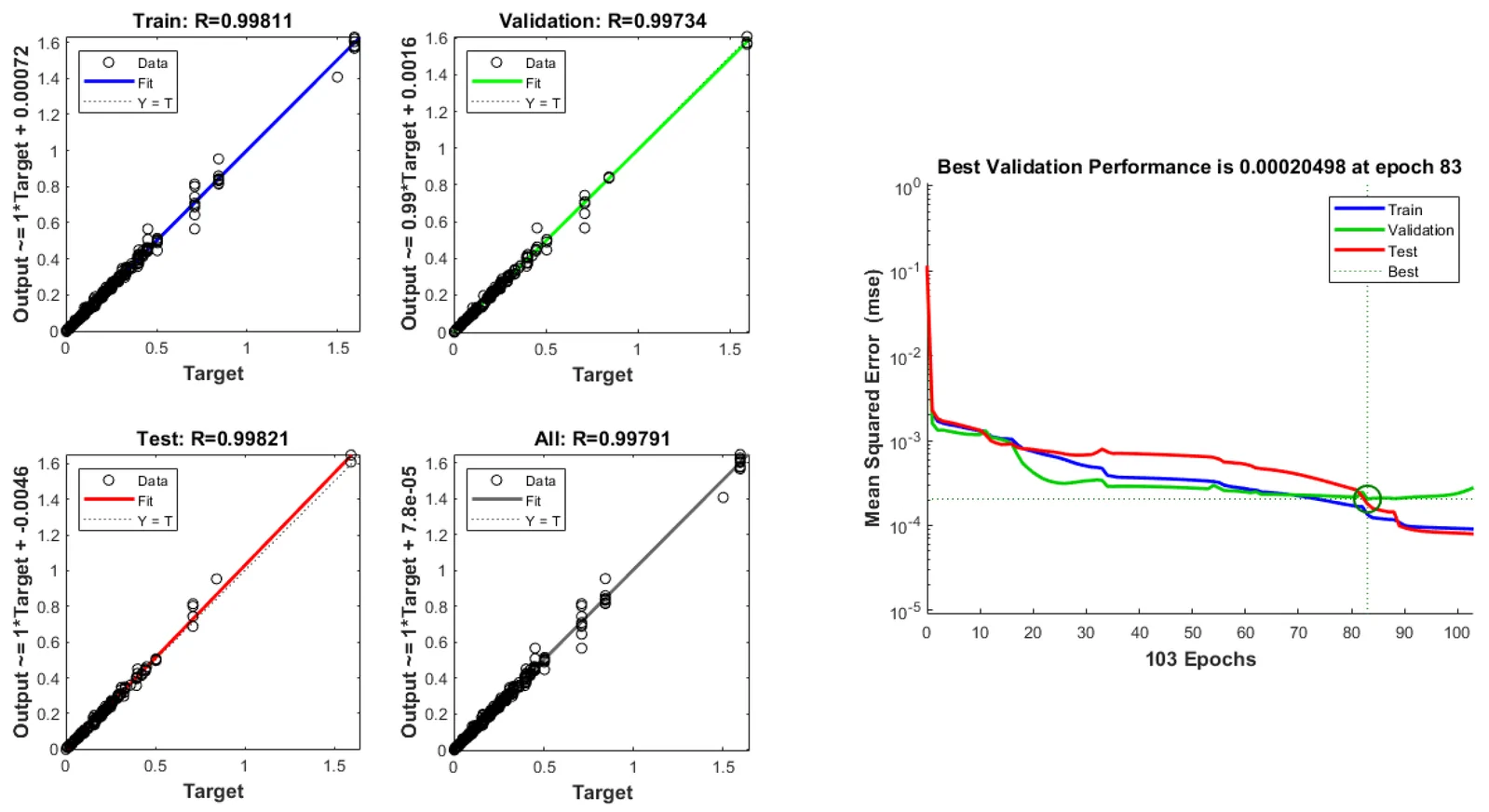
ESOA-DNN regression results.

Once the DNN topology is established, a hybrid algorithm combining ESOA and DNN techniques is utilized to optimize the initial weights and biases of DNN. After the DNN topology is determined, a hybrid algorithm integrating ESOA and DNN techniques is employed to optimize the initial weights and biases of the model. These values are optimized to enhance the accuracy of PID gain predictions, provided that the initial weights and biases are appropriately selected. To this end, the ESOA is applied in combination with the DNN technique. The weight values in the algorithm are bounded between −1 and 1. By running this hybrid approach, the optimized initial weights and biases are obtained. Then, the optimal initial weights and biases are used to fine-tune the network with Levenberg–Marquardt (LM) for the best accuracy and generalization. As a result, the performance of the DNN model based on the optimized training strategy through the real data achieves better prediction than using conventional DNN. The corresponding MSE was 0.00020498 with 83 epochs, and the regression coefficient reached 0.99791, as presented in Figure 12. All regression performance of the ESOA-DNN model is highlighted in Figure 12.

In the ESOA-CDNN workflow, the dataset was partitioned into 70% training, 15% validation, and 15% testing using the `dividerand` function. Normalization was applied to both inputs and outputs through `mapminmax` and `removeconstantrows`. To ensure reproducibility, random seeds were fixed using `rng(42,’twister’)`. The base network was trained for 250 epochs, followed by an additional 300 epochs during fine-tuning with the Levenberg–Marquardt (LM) algorithm. Stopping criteria included a minimum gradient threshold of `1 × 10^−7^` and a maximum validation failure count between 10 and 20. The ESOA optimizer employed a population size of 30 agents and 300 iterations for both architecture and weight optimization. The search bounds for hidden neurons were set between [4, 0] and [60, 60], while the bounds for weights and biases were [−1, 1]. All experiments used identical data partitions to guarantee fair comparison.

In the current study, the RMSE and MAE metrics were used to quantify the prediction errors. In addition, the R^2^ index was computed to assess the correlation between the actual and values of *K_P_*, *K_I_*, and *K_D_*. The results obtained for both training and testing stages are illustrated in Table 3.

### 3.5. PSO-CDNN PID Tuning

**Figure 13:**
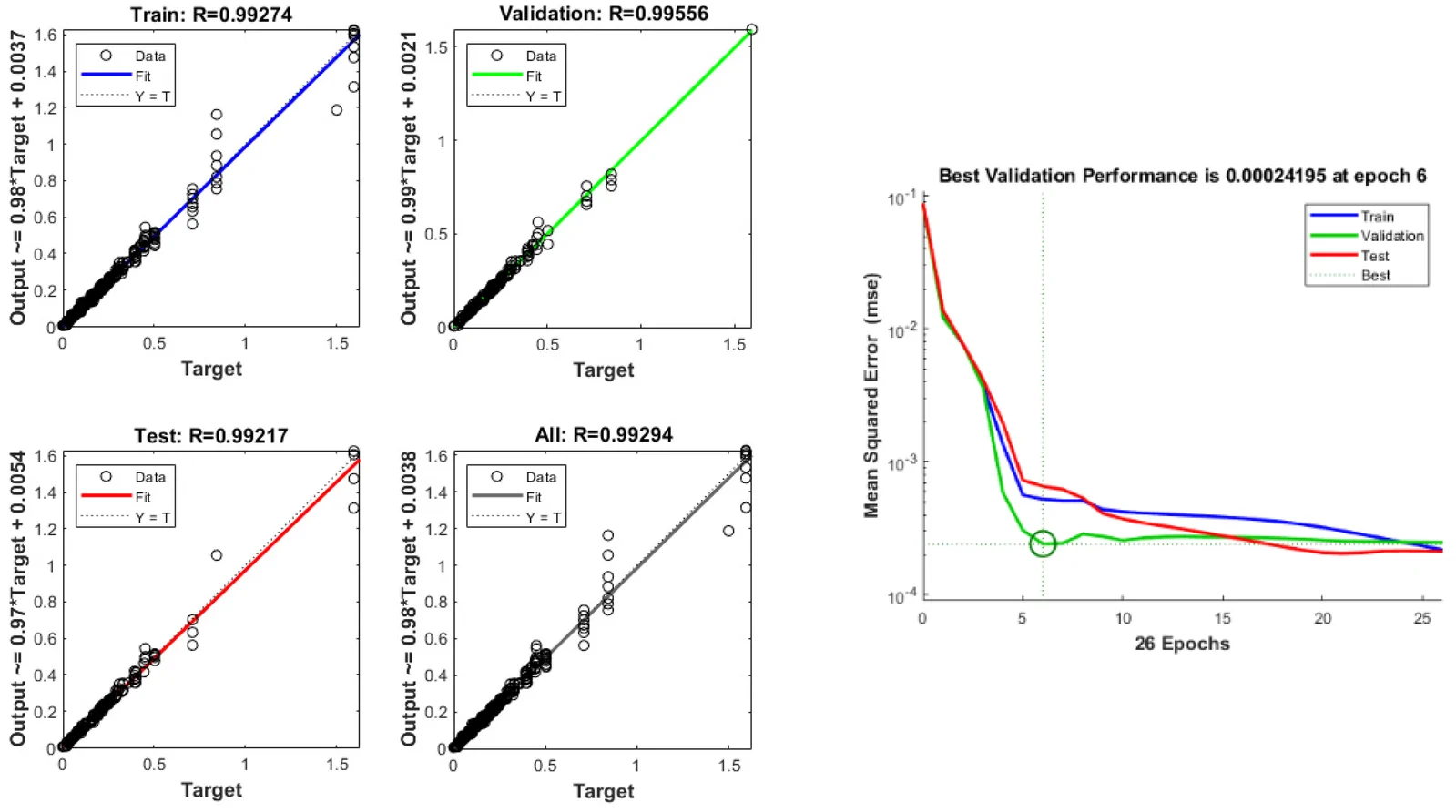
PSO-DNN regression results.

By also using another MATLAB executable code file (.m file), a DNN based on a real data set of PID tuning is proposed. Particle swarm optimization PSO was also used to improve and optimize the Deep Neural Network DNN for tuning the PID controller and compare the PSO_DNN results with the ESOA_DNN results. The same steps were taken that were done with the ESOA-CDNN. The parameters of the PSO optimizers were configured as follows: c1 = 0.8, c2 = 0.8, w = 0.9, 0.8. The optimal hidden layer obtained was 40 neurons in the first layer and 30 in the second. The DNN performance model using the PSO optimization strategy indicated superior predictions achieved compared to the conventional DNN. The corresponding MSE was 0.00024195 with six epochs, and the regression coefficient reached 0.99294, as presented in Figure 13, while Figure 11 demonstrates a schematic representation of the PSO–ANN training methodology. Table 4 shows the statistical results of PSO-DNN.

In the PSO-CDNN workflow, the same dataset partitioning (70% training, 15% validation, 15% testing) and normalization procedures (`mapminmax` and `removeconstantrows`) were applied. Random seeds were also fixed with `rng(42,’twister’)` to ensure repeatability. Training epochs were set to 250 for the base network and 300 for LM fine-tuning. Stopping criteria included `min_grad = 1 × 10^−7^` and `max_fail = 10–20`. The PSO optimizer used a population size of 30 agents and 300 iterations for both architecture and weight optimization. The bounds for hidden neurons were identical to ESOA ([4, 0] to [60, 60]), and the bounds for weights and biases were set to [−1, 1]. As with ESOA -CDNN, identical data partitions were used to ensure methodological fairness.

The variation in the number of neurons among the three networks is attributed to the different optimization approaches employed. In the first network, CDNN, the hidden-layer configuration (20–20) was manually selected after extensive trial and error to achieve the best performance, as presented in Table 2. In contrast, the second and third networks (ESOA-CDNN and PSO-CDNN) were optimized using metaheuristic algorithms. The ESOA-CDNN model identified the (6–16) configuration as providing the highest accuracy, whereas the PSO-CDNN model determined (40–30) as the optimal configuration. Therefore, the differences in neuron numbers result from the applied optimization methods rather than from arbitrary selection based on the best experimental results. The network architectures of the CDNN, PSO-CDNN, and ESOA-CDNN models are presented in Table 5.

## 4. Experimental Results and Discussion

After the four different PID tuning methods, which were described in detail in the previous sections, were applied, the PID gain values (*K_P_*, *K_I_*, and *K_D_*) obtained for each technique are presented in Table 6.

The experimental study comprised both loading and unloading scenarios. All relevant measurements were acquired via the PLC/SCADA system, and the obtained data were later processed to generate graphical representations for detailed evaluation. In order to rigorously assess the dynamic performance of the synchronous generator (SG) and associated controllers, step changes were applied instead of gradual load variations in both test cases. The test conditions included sudden application and removal of a 500 W resistive load (50%), a 1000 W resistive load (100%), and a 550 VA inductive load with a power factor of 0.8. The test loads were switched on at t = 0, resulting in a step change.

**Figure 14:**
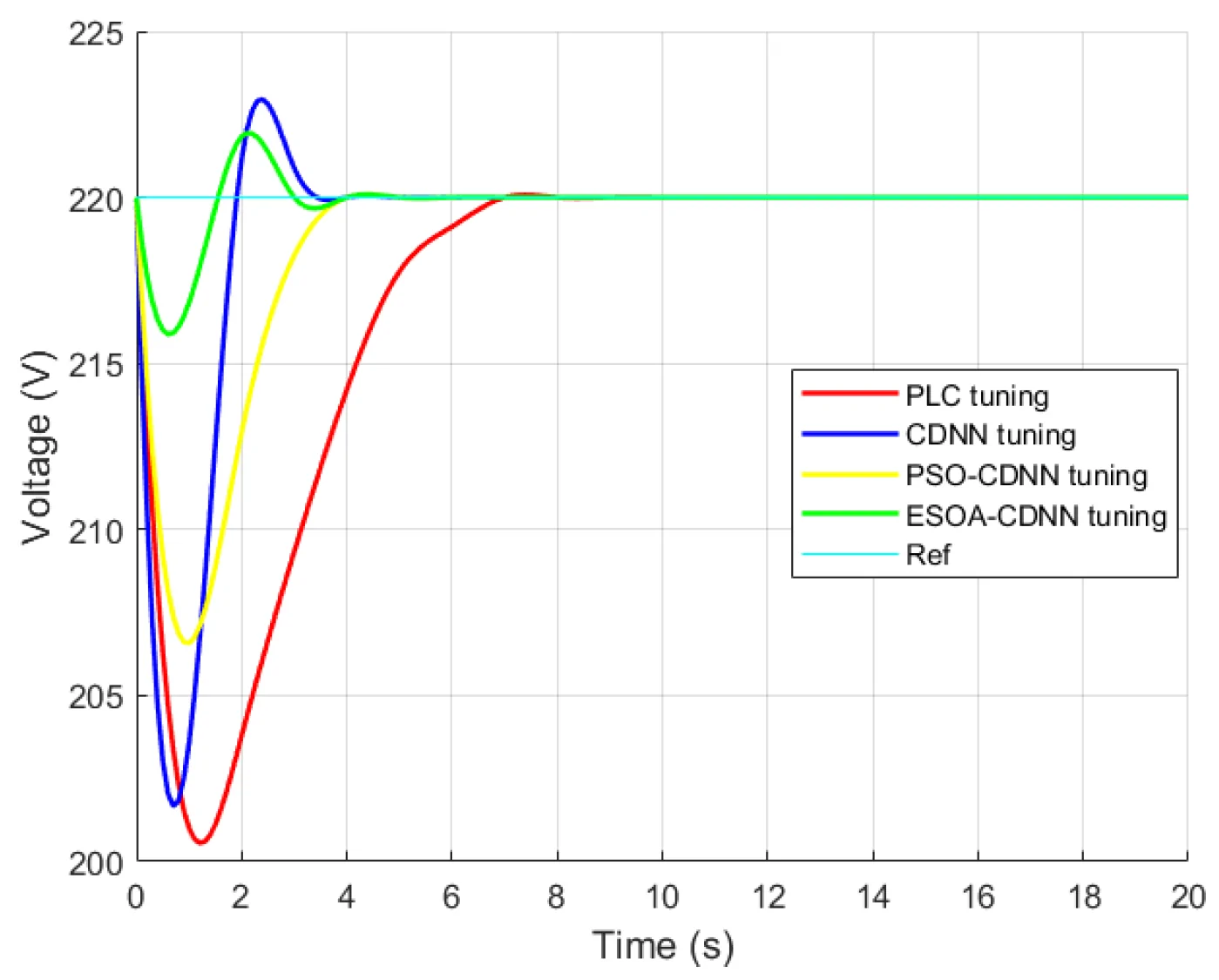
Loading experiment: 0–500 W.

**Figure 15:**
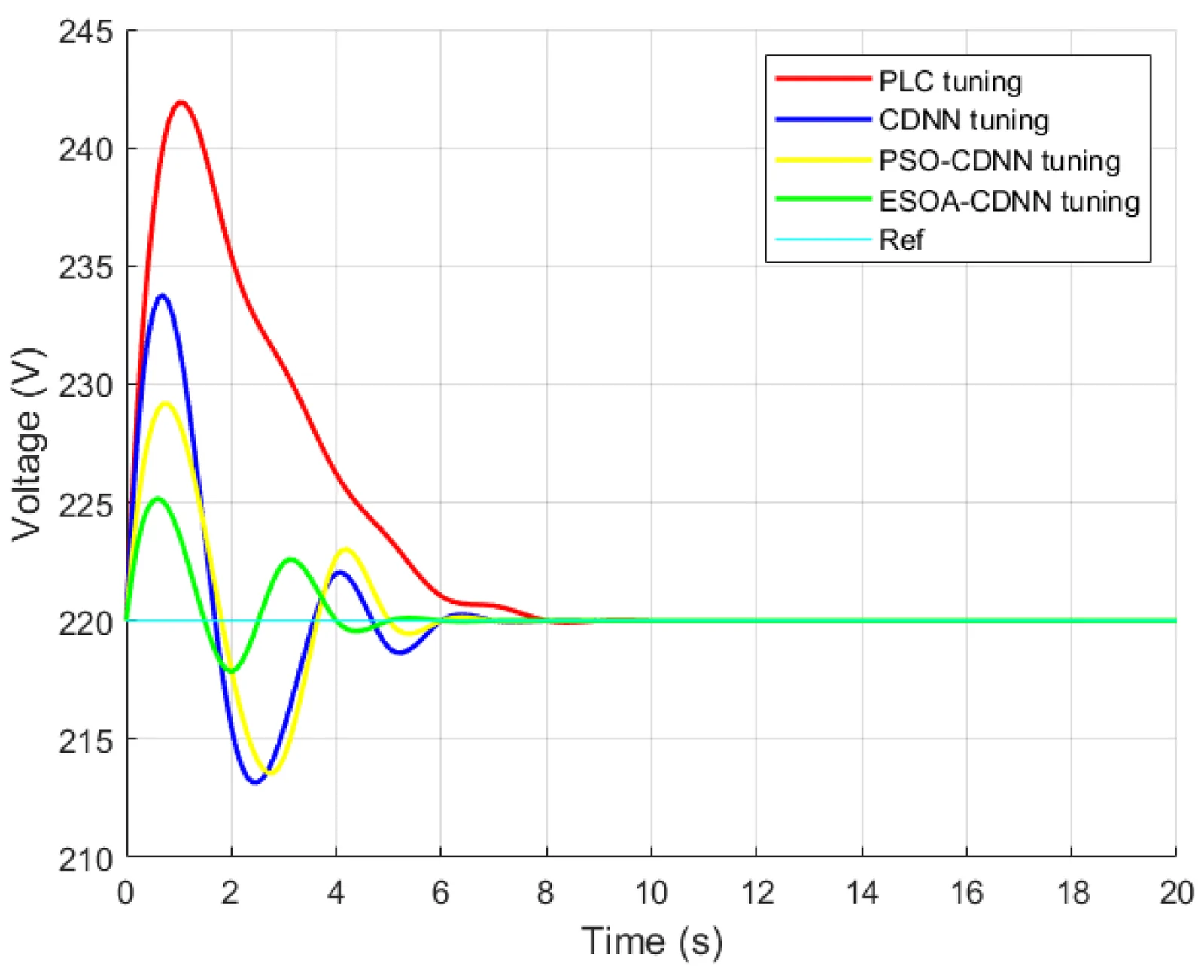
Unloading experiment: 500–0 W.

Figure 14 and Figure 15 present the findings of the experiments from no-load to 500 W load (0–50% loading) and from 500 W load back to unloading, respectively. In the 500 W instantaneous loading test, the minimum voltage values observed with the PLC-PID, CDNN-PID, PSO-CDNN PID, and ESOA-CDNN PID controllers, were 200.1 V (maximum undershoot 19.9 V), 201.7 V (18.3 V), 206.6 V (13.4 V) and 215.9 V (4.1 V), respectively. The settling times were 7 s, 4 s, 4 s, and 3 s for PLC-PID, CDNN-PID, PSO-CDNN PID, and ESOA-CDNN PID controllers, respectively. In the 500 W instantaneous unloading experiment, the maximum voltage values recorded for PLC-PID, CDNN-PID, PSO-CDNN PID, and ESOA-CDNN PID controllers were 241.9 V (with a maximum overshoot of 21.9 V), 233.6 V (with a maximum overshoot of 13.6 V), 229.2 V (with a maximum overshoot of 9.2 V) and 225.1 V (with a maximum overshoot of 5.1 V), respectively. The settling times for PLC-PID, DNN-PID, PSO-DNN PID, and ESOA-DNN PID controllers were 8 s, 6 s, 5 s, and 4 s, respectively.

**Figure 16:**
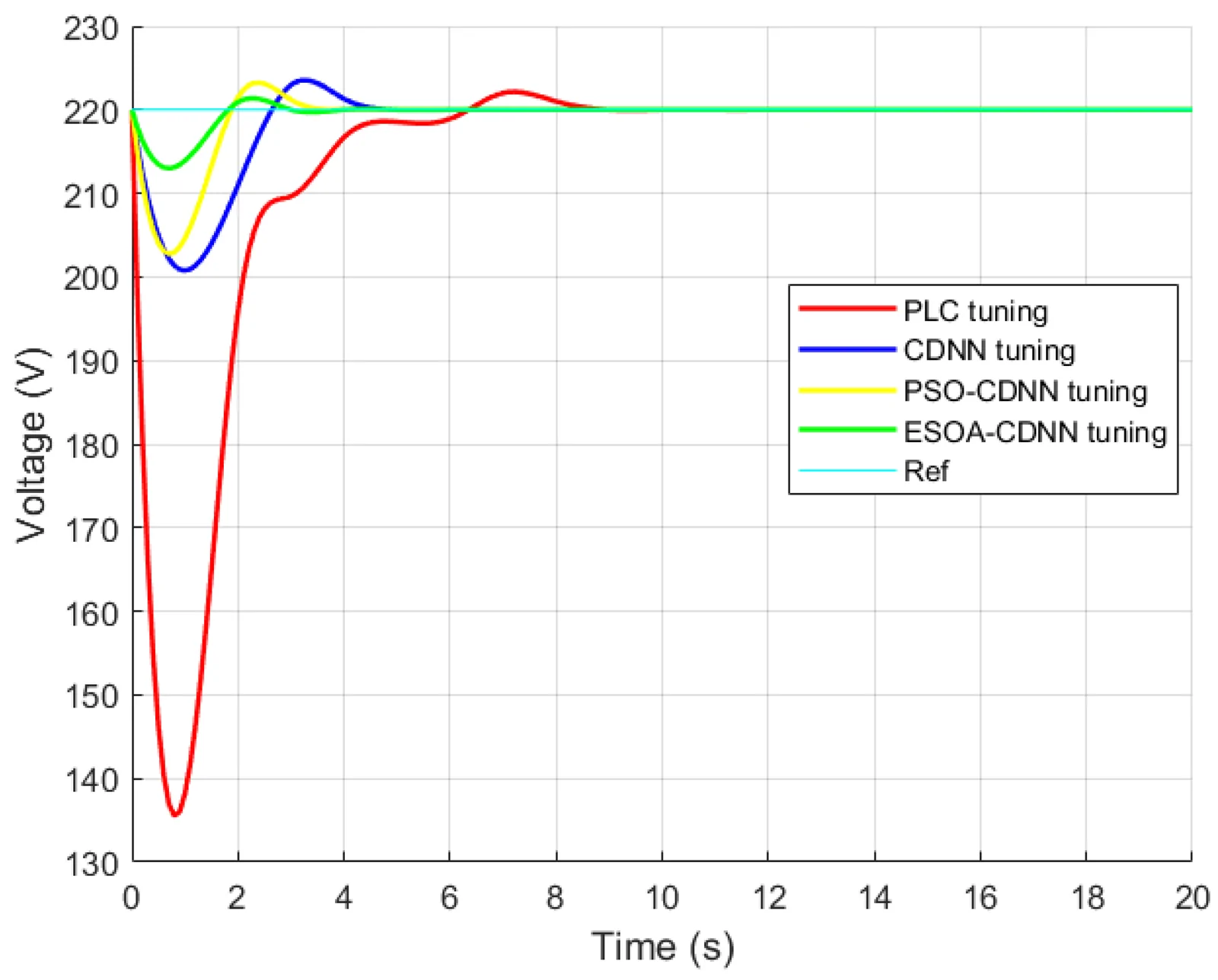
Loading experiment: 0–1000 W.

**Figure 17:**
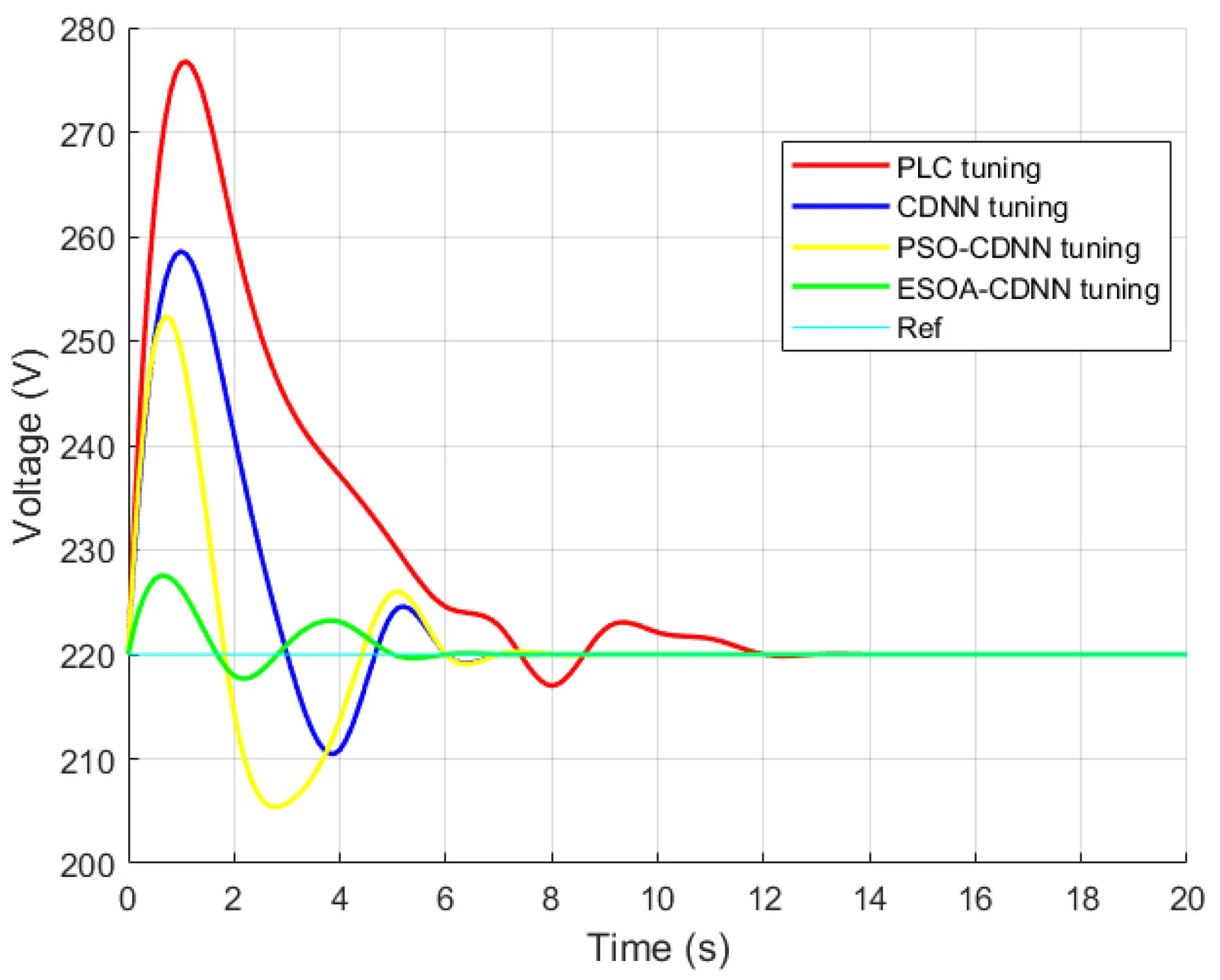
Unloading experiment: 1000–0 W.

The findings of the no-load to 1000 W load (0–100% loading) and 1000 W load to no-load (unloading) experiments are presented in Figure 16 and Figure 17, respectively. In the test using the conventional PID (PLC-PID) controller, the minimum voltage observed was 135.5 V, with a maximum undershoot of 84.5 V and a settling time of 10 s. With the CDNN-PID controller, the minimum voltage increased to 200.8 V, the maximum decreased to 19.2 V and the settling time was reduced to 5 s. For the PSO-CDNN PID controller, the minimum voltage reached 202.8 V, the maximum undershoot was 17.2 V, and the settling time was 4 s. The ESOA-CDNN PID controller achieved the best performance with a minimum voltage 213.1 V, a maximum undershoot of only 6.9 V, and a settling time of 3 s. In the 1000 W unloading experiment, the maximum voltage values were 276.7 V (maximum overshoot of 56.7 V) for the PLC-PID controller, 258.6 V (maximum overshoot of 37.4 V) for the CDNN-PID controller, 252.4 V (maximum overshoot of 32.4 V) for the PSO-CDNN PID controller, and 227.5 V (maximum overshoot of 7.5 V) for the ESOA-CDNN PID controllers. The corresponding settling times were 12 s for the PLC-PID, 6 s for both the CDNN PID and PSO-CDNN PID, and 5 s for the ESOA-CDNN PID controller.

**Figure 18:**
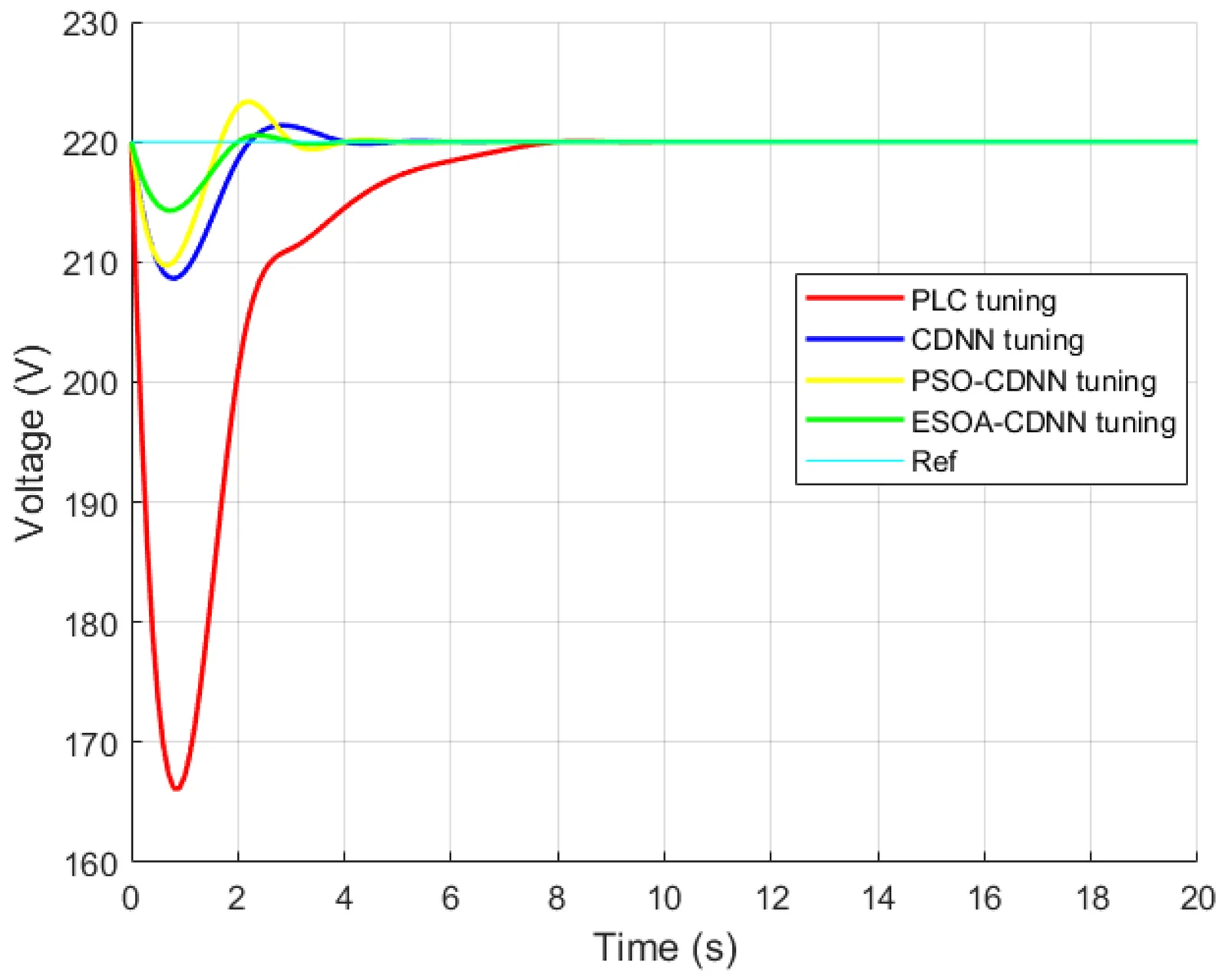
Loading experiment: 0–550 VA.

**Figure 19:**
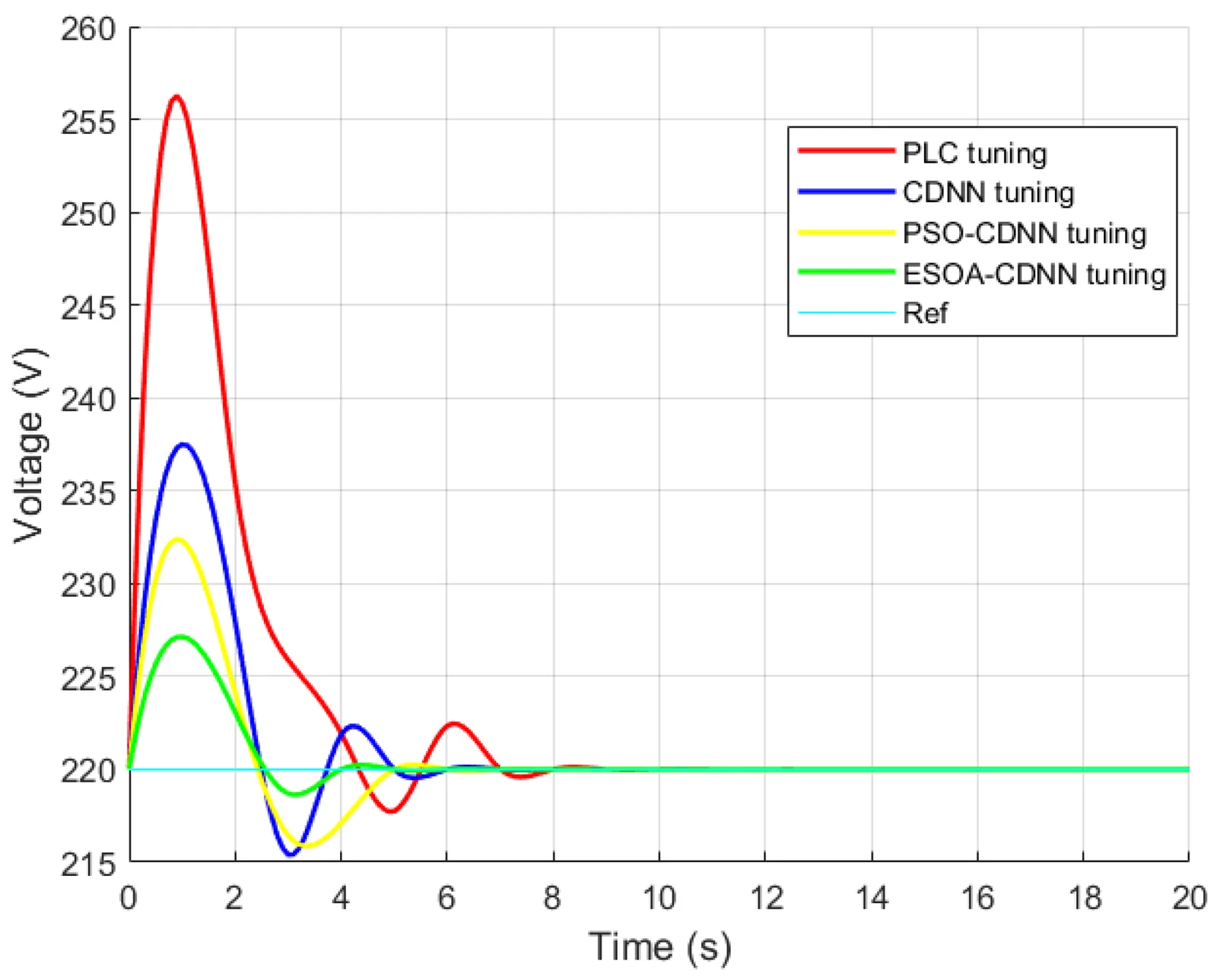
Unoading experiment: 550–0 VA.

The findings of no-loading to 550 VA (power factor = 0.8) load and 550 VA load to unloading experiments, are shown in Figure 18 and Figure 19, respectively. In the 550 VA instantaneous loading test, the minimum voltage values observed for the PLC-PID, CDNN-PID, PSO-CDNN PID, and ESOA-CDNN PID controllers were 166.1 V (maximum undershoot of 53.9 V), 208.6 V (maximum undershoot of 11.4 V), 209.7 V (maximum undershoot of 10.3 V), and 214.3 V (maximum undershoot of 5.7 V), respectively. The corresponding settling times were 8 s, 4 s, 3 s, and 2 s for PLC-PID, CDNN-PID, PSO-CDNN PID, and ESOA-CDNN PID controllers, respectively. In the 550 VA (power factor = 0.8) instantaneous unloading experiment, the maximum voltage values recorded for the PLC-PID, CDNN-PID, PSO-CDNN PID, and ESOA-CDNN PID controllers were 256.3 V (maximum overshoot of 36.3 V), 237.5 V (maximum overshoot of 17.5 V), 232.3 V (maximum overshoot of 12.3 V), and 227.1 V (maximum overshoot of 7.1 V), respectively. The corresponding settling times were 7 s, 5 s, 5 s, and 4 s for the PLC-PID, CDNN-PID, PSO-CDNN PID, and ESOA-CDNN PID controllers, respectively.

The findings for the PLC-PID, CDNN-PID, PSO-CDNN PID, and ESOA-CDNN PID tuning are given in Table 7 and Table 8 for the loading and unloading experiments, respectively. To evaluate the repeatability of the experimental results, all loading/unloading tests were repeated seven times under identical operating conditions. The obtained results are presented as mean ± standard deviation in Table 7 and Table 8. No steady-state error was observed in any of the experiments.

The experimental findings indicated that the PID controller tuned by ESOA-CDNN outperforms the tuned by PLC, CDNN and PSO-CDNN PID controller. The comparison of the loading and unloading test results demonstrates that the PID tuned by ESOA-DNN provides a substantial improvement in overall system performance when compared with the tuned by PLC, CDNN and PSO-CDNN, in particular lower overshoot.

Loading and unloading (load shedding) tests in the experimental study were carried out under demanding operating conditions, involving abrupt and large step changes in load at 50% and 100% (resistive load) and 55% (inductive load) of the system nominal power. Such instantaneous variations at these magnitudes are rarely encountered in practical power systems, where load changes typically occur more gradually. Under smaller or more incremental load variations, for example 10% or 20% of nominal power, the output voltage for all four tuning methods was able to reach the reference value within approximately 1 s or less.

## 5. Conclusions

In this study, a novel ESOA-DNN PID algorithm was developed for tuning the PID controller in the Automatic Voltage Regulator (AVR) system of a stand-alone synchronous generator, and its performance was validated using real industrial hardware under practical operating conditions. The proposed algorithm effectively optimized CDNN parameters. Experimental results demonstrated that the proposed model significantly reduced settling time and overshoot compared to PID controller tuned by conventional PLC while ensuring robust and stable voltage regulation under varying load conditions. In the 0–1000 W loading experiment, the settling times for the PLC, CDNN, PSO-CDNN, and ESOA-CDNN tuning methods were 10 s, 5 s, 4 s, and 3 s, respectively, while the maximum undershoots were 84.5 V (38.4%), 19.2 V (8.72%), 17.2 V (7.81%), and 6.9 V (3.13%), respectively. All experimental results were presented in Figure 14, Figure 15, Figure 16, Figure 17, Figure 18 and Figure 19 and Table 7 and Table 8. This highlights the practical applicability and theorical relevance of the algorithm for industrial and academic applications. The real-time control and monitoring of the system were implemented using an S7-1200 PLC in conjunction with the TIA Portal V17 software environment. This study has demonstrated that the proposed metaheuristic algorithm can be effectively deployed on a shallow industrial controller platform such as a PLC. Optimal values of the PID gains $K_{P}$, $K_{I}$, and $K_{D}$ were obtained for the control of the nonlinear AVR system under various loading conditions.

Future work will investigate the performance of the proposed framework against additional machine learning-based regression models and compare ESOA-CDNN with the latest state-of-the-art AVR methods using the same benchmark datasets and hardware settings.

## Figures and Tables

**Table 1:** Experimental parameters and real-time implementation details of the PLC-based AVR System.

Compenent/Parameter	Future/Function
SG	3 phase, 4 wired, 1 kW, 380 V, 2.3 A AC, 1500 rpm, brushed, Exct voltage: 72 V DC, Exct current: 2.1 A DC.
Energy Meter Module (SM1238)	Voltages, Currents, Phase angels, Power, Frequencies, Power factor. Adres space: 124 byte; 112 byte input/12 byte output. Cycle time (all channels), typ. 50 ms; Time for consistent update of all measured and calculated values (cyclic und acyclic data)
PLC Analog Output (SM1234)	0–10 V DC, 14 bit.
DC-DC Converter	AI: 0–10 V DC, 13 bit, Output Voltage: 0–90 V DC, Output Current: 15 A DC.
PLC Cycle Time	Shortest cycle time: 1 ms, Current/last cycle time: 4 ms, Longest cycle time: 8 ms.
Sampling Time of PID Algorithm	12 ms.
Load-Switching Timing	The test loads were switched on at t = 0, resulting in a step change.

**Table 2:** DNN architectures and performance results.

Training No.	ANN Architecture	Training Regression	MSE
Net.1	4 × 20 × 20 × 3	0.98598	0.0017782 × 10^−2^
Net.2	4 × 18 × 19 × 3	0.974	0.0021431 × 10^−2^
Net.3	4 × 10 × 10 × 3	0.94105	0.004536 × 10^−2^
Net.4	4 × 8 × 6 × 3	0.9598	0.004476 × 10^−2^
Net.5	4 × 15 × 10 × 3	0.95671	0.008780 × 10^−2^
Net.6	4 × 6 × 16 × 3	0.93879	0.009309 × 10^−2^
Net.7	4 × 10 × 5 × 3	0.92408	0.008679 × 10^−1^

**Table 3:** ESOA-CDNN statistical results.

Regression Model		Training Dataset			Validation Dataset			Test Dataset		
		RMSE	MAE	R2	RMSE	MAE	R2	RMSE	MAE	R2
ESOA	***K_P_***	0.0087656	0.0061542	0.99355	0.011426	0.0075069	0.98946	0.0090817	0.0064644	0.99216
	***K_I_***	0.017553	0.0091047	0.9946	0.021686	0.010542	0.99266	0.020893	0.009636	0.99268
	***K_D_***	0.0042783	0.0030804	0.99441	0.0037534	0.0025623	0.9958	0.0044211	0.0029904	0.99338

**Table 4:** PSO-CDNN statistical result.

Regression Model		Training Dataset			Validation Dataset			Test Dataset		
		RMSE	MAE	R2	RMSE	MAE	R2	RMSE	MAE	R2
PSO	***K_P_***	0.010882	0.0078404	0.99002	0.0116	0.0082019	0.98766	0.0094841	0.0070313	0.99263
	***K_I_***	0.037692	0.014617	0.97619	0.023622	0.012669	0.98512	0.042927	0.018641	0.97507
	***K_D_***	0.0056515	0.0042152	0.9902	0.0057711	0.0041603	0.98957	0.0058942	0.0045468	0.98911

**Table 5:** Architecture of the proposed neural network models.

Network	Hidden Layers	Neurons per Layer	Optimization Method
CDNN	2	20,20	Trial and error
ESOA-CDNN	2	6,16	ESOA
PSO-CDNN	2	40,30	PSO

**Table 6:** *K_P_*, *K_I_*, and *K_D_* values.

Gains	PLC PID	CDNN PID	PSO-CDNN PID	ESOA-CDNN PID
***K_P_***	0.1034	0.6709	0.5585	0.1829
***K_I_***	0.2605	1.0772	0.3261	0.2067
***K_D_***	0.0620	0.0579	0.0408	0.0654

**Table 7:** PLC, CDNN, PSO-CDNN and ESOA-CDNN PID tuning comparison for loading experiments.

Experiment	Settling Time (s)				Max. Undershoot (V)			
	PLC	CDNN	PSO-CDNN	ESOA-CDNN	PLC	CDNN	PSO-CDNN	ESOA-CDNN
0–500 W	7 ± 2	4 ± 1	4 ± 1	3 ± 1	19.9 (9.04 ± 1.8%)	18.3 (8.31 ± 1.5%)	13.4 (6.09 ± 1.2%)	4.1 (1.86 ± 0.3%)
0–1000 W	10 ± 2	5 ± 1	4 ± 1	3 ± 1	84.5 (38.4 ± 2.1%)	19.2 (8.72 ± 1.4%)	17.2 (7.81 ± 1.1%)	6.9 (3.13 ± 0.3%)
0–550 VA	8 ± 1	4 ± 1	3 ± 1	2 ± 1	53.9 (24.5 ± 1.7%)	11.4 (5.18 ± 1.2%)	10.3 (4.68 ± 1%)	5.7 (2.59 ± 0.2%)

**Table 8:** PLC, CDNN, PSO-CDNN and ESOA-CDNN PID tuning comparison for unloading experiments.

Experiment	Settling Time (s)				Max. Overshoot (V)			
	PLC	CDNN	PSO-CDNN	ESOA-CDNN	PLC	CDNN	PSO-CDNN	ESOA-CDNN
500–0 W	8 ± 1	6 ± 1	5 ± 1	4 ± 1	21.9 (9.95 ± 1.5%)	13.6 (6.18 ± 1.4%)	9.2 (4.18 ± 1.1%)	5.1 (2.31 ± 0.1%)
1000–0 W	12 ± 2	6 ± 1	6 ± 1	5 ± 1	56.7 (25.77 ± 2.5%)	38.6 (17.54 ± 1.7%)	32.4 (14.72 ± 1.3%)	7.5 (3.41 ± 0.3%)
550–0 VA	7 ± 1	5 ± 1	5 ± 1	4 ± 1	36.3 (16.5 ± 1.7%)	17.5 (7.95 ± 1.1%)	12.3 (5.59 ± 1.2%)	7.1 (3.22 ± 0.1%)

## Data Availability

All data supporting the findings of this study are included within the manuscript.
